# Supplementary material for: Investigating the association between genetically proxied circulating levels of immune checkpoint proteins and cancer survival: protocol for a Mendelian randomisation analysis
Source: BMJ Open. 2024 Feb 15;14(2):e075981. doi: 10.1136/bmjopen-2023-075981 (PMC10875531; doi:10.1136/bmjopen-2023-075981)
Supplement: Supplementary data [file bmjopen-2023-075981supp001.pdf]

SUPPLEMENTARY MATERIAL

Supplementary table 1: Medicines and Healthcare products Regulatory Agency indications of anti-programmed cell death protein 1 (PD-1) and anti-programmed cell death ligand 1 (PD-L1) monoclonal antibodies obtained 25th March 2023 (* represents monotherapy indications and ** represents indications as part of combination therapy).
Molecular markers: epidermal growth factor receptor gene (*EGFR*), anaplastic lymphoma kinase gene (*ALK*), c-ros oncogene 1 gene (*ROS1*), deficient mismatch repair (dMMR), microsatellite instability high (MSI-H), combined positive score (CPS), tumour proportion score (TPS).
Cancers: non-small cell lung cancer (NSCLC), head and neck squamous cell carcinoma (HNSCC), triple-negative breast cancer (TNBC), small cell lung cancer (SCLC).

Target	Drug	Cancer						
		Breast	Colorectal	Head and neck	Lung	Melanoma	Ovarian	Prostate
PD-1	Cemiplimab (13)	-	-	-	• Metastatic or locally advanced PD-L1 ≥ 50% TPS NSCLC (*EGFR*, *ALK*, *ROS1* wildtype)*	-	-	-
	Dostarlimab (14)	-	-	-	-	-	-	-
	Nivolumab (15)	-	• dMMR/MSI-H colorectal cancer following chemotherapy**	• Metastatic or recurrent HNSCC following prior treatment*	• Metastatic NSCLC (*EGFR*, *ALK* wild type)**<br>• Metastatic or locally advanced NSCLC following chemotherapy*<br>• Resectable NSCLC**	• Metastatic or unresectable melanoma*,**<br>• Metastatic or lymph node involved melanoma patients following complete resection*	-	-

	Pembrolizumab (16)	• Locally advanced or early-stage TNBC with high risk of recurrence*,**<br>• Metastatic or locally recurrent unresectable PD-L1 CPS ≥ 10 TNBC**	• Treatment-naïve metastatic dMMR/MSI-H colorectal cancer*<br>• Metastatic or unresectable dMMR/MSI-H colorectal cancer following prior treatment*	• Treatment-naïve metastatic or unresectable recurrent PD-L1 CPS ≥ 1 HNSCC*,**<br>• Metastatic or recurrent PD-L1 TPS ≥ 50% HNSCC following prior chemotherapy*	• Metastatic NSCLC with PD-L1 ≥ 50% TPS (*EGFR*, *ALK* wild-type)*,**<br>• Metastatic or locally advanced PD-L1 ≥ 1% TPS NSCLC following chemotherapy and targeted therapy (if applicable)*	•Metastatic or unresectable melanoma*<br>•Completely resected stage IIB, IIC, III melanoma*	-	-
PD-L1	Atezolizumab (17)	• Metastatic or locally advanced PD-L1 ≥ 1% TPS TNBC**	-	-	• Completely resected stage II-IIIA NSCLC with PD-L1 ≥ 50% and no progression on prior chemotherapy*<br>• Metastatic nonsquamous NSCLC**<br>• Metastatic NSCLC PD-L1 ≥ 50% TPS (*EGFR*, *ALK* wildtype)*<br>• Metastatic or locally advanced NSCLC following chemotherapy and targeted therapy (if applicable)*<br>• Treatment-naïve extensive stage SCLC**	-	-	-
	Avelumab (18)	-	-	-	-	-	-	-

	Durvalumab (19)	-	-	-	• Locally advanced unresectable PD-L1 ≥ 1% TPS NSCLC without progression on chemoradiation*<br>• Treatment-naïve extensive-stage SCLC**	-	-	-

Bate T, *et al. BMJ Open* 2024; 14:e075981. doi: 10.1136/bmjopen-2023-075981
