# Supplementary material for: Investigating the association between genetically proxied circulating levels of immune checkpoint proteins and cancer survival: protocol for a Mendelian randomisation analysis
Source: BMJ Open. 2024 Feb 15;14(2):e075981. doi: 10.1136/bmjopen-2023-075981 (PMC10875531; doi:10.1136/bmjopen-2023-075981)
Supplement: Author's manuscript [file bmjopen-2023-075981.draft_revisions.pdf]

BMJ Open

BMJ Open is committed to open peer review. As part of this commitment we make the peer review history of every article we publish publicly available.

When an article is published we post the peer reviewers' comments and the authors' responses online. We also post the versions of the paper that were used during peer review. These are the versions that the peer review comments apply to.

The versions of the paper that follow are the versions that were submitted during the peer review process. They are not the versions of record or the final published versions. They should not be cited or distributed as the published version of this manuscript.

BMJ Open is an open access journal and the full, final, typeset and author-corrected version of record of the manuscript is available on our site with no access controls, subscription charges or pay-per-view fees (http://bmjopen.bmj.com).

If you have any questions on BMJ Open's open peer review process please email info.bmjopen@bmj.com

BMJ Open

Protocol for a Mendelian randomisation analysis investigating the association between genetically proxied circulating levels of immune checkpoint proteins and cancer survival.

Journal:	*BMJ Open*
Manuscript ID	bmjopen-2023-075981
Article Type:	Protocol
Date Submitted by the Author:	24-May-2023

Complete List of Authors:	Bate, Tessa; University of Bristol<br>Martin, Richard; University of Bristol, Social Medicine<br>Yarmolinsky, James ; University of Bristol,<br>Haycock, Philip; Bristol University

Keywords:	ONCOLOGY, Cancer genetics < GENETICS, Prognosis, THERAPEUTICS

SCHOLARONE™
Manuscripts

Protocol for a Mendelian randomisation analysis investigating the association between genetically proxied circulating levels of immune checkpoint proteins and cancer survival.

Tessa Bate[1,2], Richard M Martin[1-3], James Yarmolinsky [1,2]*, Philip C Haycock [1,2]*

[1] Medical Research Council Integrative Epidemiology Unit, University of Bristol
[2] Population Health Sciences, Bristol Medical School, University of Bristol
[3] NIHR Bristol Biomedical Research Centre, University Hospitals Bristol and Weston NHS Foundation Trust and the University of Bristol

Corresponding author:
Tessa Bate, MSc
MRC Integrative Epidemiology Unit,
University of Bristol
oi22026@bristol.ac.uk

For peer review only

ABSTRACT

Introduction

Compared to the traditional drug development pathway, investigating alternative uses for existing drugs (i.e., drug repurposing) requires substantially less time, cost, and resources. Immune checkpoint inhibitors are licensed for the treatment of certain breast, colorectal, lung and melanoma cancers. These drugs target immune checkpoint proteins to reduce the suppression of T cell activation by cancer cells, enabling normal T cell activation. As T cell suppression is a hallmark of cancer common across sites anatomical sites, we hypothesise that immune checkpoint inhibitors could be repurposed for the treatment of additional cancers to the ones already indicated.

Methods and analysis

We will use two-sample Mendelian randomisation to investigate the effect of genetically proxied expression of the protein targets of two immune checkpoint inhibitors - programmed cell death protein 1 (PD-1) and programmed death ligand 1 (PD-L1) - on survival of six cancer types (breast, colorectal, lung, melanoma, ovarian, and prostate).

Summary genetic association data will be obtained from prior genome-wide association studies of circulating protein expression and cancer survival in populations of European ancestry. Various sensitivity analyses will be performed to examine the robustness of findings to potential violations of Mendelian randomisation assumptions and the impact of alternative genetic instrument construction strategies. The impact of treatment history and tumour stage on the findings will also be investigated using summary-level and individual-level genetic data where available.

Ethics and dissemination

No separate ethics approval will be required for these analyses as we will be using data from previously published genome-wide association studies which individually gained ethical approval and participant consent. Results from analyses and statistical code will be made freely available upon the completion of the analysis.

STRENGTHS AND LIMITATIONS OF THIS STUDY

- As germline genetic variants proxying circulating protein levels are randomly assorted at meiosis and fixed at conception, Mendelian randomisation analyses examining the effect of these proteins on cancer survival should be less prone to conventional issues of confounding and cannot be influenced by reverse causation bias.
- The use of a two-sample Mendelian randomisation framework will permit us to leverage large-scale genetic association data from separate samples, thus enhancing statistical power and precision of estimates.
- The generalisability of our findings to populations of non-European ancestry may be unclear.
- Mendelian randomisation analysis can only evaluate the on-target effects of immune checkpoint inhibitors.

For peer review only

INTRODUCTION

Drug repurposing is the use of approved drugs for another indication (1, 2). The traditional development and testing pathway of candidate drugs is expensive and time-consuming, with an estimated cost of $2-3 billion USD and 13 years of research on average required for a chemical compound to be approved for use in clinical practice (3). In contrast, drugs that are tested for a repurposed use should already have demonstrated success in phase I trials for their original indication and thus their safety profiles for human use are known (3-6). Consequently, clinical testing for a repurposed use of a drug can begin at phase II trials, reducing associated time and resource requirements (3-6).

Despite advances in screening and treatment strategies, the number of people diagnosed with, and dying from cancer, continues to increase. Globally, there were estimated to be 19.3 million new cancer diagnoses and 10.0 million cancer deaths in 2020 (7). Six cancer sites (breast, colorectal, lung, melanoma skin, ovarian, and prostate cancer) were estimated to contribute to 44% of the incidence and 41% of mortality from all cancer sites globally in 2020 (7). In addition to the high burden of cancer, there are issues associated with currently available treatments such as development of resistance, severity of side effects, and lack of efficacy in some individuals (8). Identifying new strategies for the treatment of these high-burden cancers using drug repurposing could minimise the cost and patient involvement required for the assessment of their efficacy. Several drugs have been successfully repurposed for cancer treatment, including non-cancer drugs such as thalidomide which was originally developed to treat morning sickness in pregnancy but is now approved to treat multiple myeloma (3, 8, 9).

Shared hallmarks of cancer common to different cancer sites represent an opportunity for drug repurposing using approved drugs which target these mechanisms across multiple sites (3). One such hallmark is the avoidance of immune destruction, which can be suppressed using immune checkpoint inhibitors (3, 10, 11). The first immune checkpoint inhibitor approved by the US Food and Drug Administration (FDA) was the anti-cytotoxic T-lymphocyte associated protein 4 (CTLA-4) monoclonal antibody, ipilimumab, for the treatment of melanoma in 2011 (10). Following the approval of ipilimumab, several other immune checkpoint inhibitors have also been approved for a range of cancer indications.

Two examples of immune checkpoint proteins which have been successfully targeted in cancer treatment are programmed cell death protein 1 (PD-1) and programmed cell death ligand 1 (PD-L1) (10). The interaction between PD-L1 on the surface of cancer cells and PD-1 on the surface of activated T cells suppresses further T cell activation (10, 12-14). The anti-PD-1 monoclonal antibodies cemiplimab (Libtayo), dostarlimab (Jemperli), nivolumab (Opdivo) and pembrolizumab (Keytruda), and the anti-PD-L1 monoclonal antibodies atezolizumab (Tecentriq), avelumab (Bavencio) and durvalumab (Imfinzi) inhibit this interaction and so enable normal T cell activation during anti-cancer immune responses (10, 15). These seven immune checkpoint inhibitors have been approved by the Medicines and Healthcare products Regulatory Agency (MHRA) for specific cancer indications, including some indications for the six cancer types detailed above (Table 1, Supplementary Table 1). Across anti-PD-1 immune checkpoint inhibitors, there are approved indications for the treatment of breast, colorectal, and lung cancers and melanoma, whilst anti-PD-L1 immune checkpoint inhibitors have been approved for breast and lung cancer treatment (16-22) (Table 1, Supplementary Table 1).

For peer review only - http://bmjopen.bmj.com/site/about/guidelines.xhtml

Table 1: Medicines and Healthcare products Regulatory Agency (MHRA) indications of anti-programmed cell death protein 1 (PD-1) and anti-programmed cell death ligand 1 (PD-L1) monoclonal antibodies obtained 25th March 2023 (A represents immune checkpoint inhibitors with at least one approved indication for the cancer type either as monotherapy or as part of combination therapy).

**Protein target**	**Immune checkpoint inhibitor**	**Cancer**					
		**Breast**	**Colorectal**	**Lung**	**Melanoma**	**Ovarian**	**Prostate**
PD-1	Cemiplimab (16)	-	-	A	-	-	-
	Dostarlimab (17)	-	-	-	-	-	-
	Nivolumab (18)	-	A	A	A	-	-
	Pembrolizumab (19)	A	A	A	A	-	-
PD-L1	Atezolizumab (20)	A	-	A	-	-	-
	Avelumab (21)	-	-	-	-	-	-
	Durvalumab (22)	-	-	A	-	-	-

For peer review only

Rather than investigating the efficacy of these specific immune checkpoint inhibitor compounds, their on-target effects will be proxied using genetic instruments which represent decreased circulating levels of their protein targets, PD-1 and PD-L1. Consequently, this study will use Mendelian randomisation (MR) to investigate the association between genetically proxied PD-1 or PD-L1 expression levels and survival of six cancer types: breast, colorectal, lung, melanoma, ovarian, and prostate cancer. These six cancer sites have been chosen for inclusion as they have the most well-powered and accessible genome-wide association study (GWAS) survival data and make an important contribution to the overall global cancer burden.

The approvals of anti-PD-1 and anti-PD-L1 immune checkpoint inhibitors are not uniform across cancer sites, even for the drugs targeting the same immune checkpoint protein (Table 1). For example, pembrolizumab is the only anti-PD-1 immune checkpoint inhibitor approved for a breast cancer indication by the MHRA (19) (Table 1), likely due to lack of complete late-stage clinical trials with large sample sizes investigating the efficacy of the other three anti-PD-1 immune checkpoint inhibitors (13, 14). Similarly, although nivolumab and pembrolizumab both have colorectal cancer indications, there are differences in the characteristics of the patient populations that they are approved to treat (18, 19) (Table 1, Supplementary Table 1). Nivolumab is approved to treat mismatch repair deficient (dMMR) or microsatellite instability high (MSI-H) colorectal cancer patients after chemotherapy as part of a combination therapy with ipilimumab (18) (Supplementary Table 1). Whereas, pembrolizumab as monotherapy is approved to treat metastatic or unresectable dMMR/MSI-H colorectal cancer, the latter following previous treatment (19) (Supplementary Table 1).

The approved indications of these immune checkpoint inhibitors are highly specific in many cases, particularly with respect to molecular tumour markers and treatment history. For example, durvalumab has been approved by the MHRA for the treatment of adult patients with locally advanced, unresectable non-small cell lung cancer if at least 1% of their tumour cells express PD-L1 and their disease did not advance after previous platinum-based chemoradiotherapy (22) (Supplementary Table 1). Our planned analyses will enable study of the potential efficacy of these immune checkpoint inhibitors in broader cancer populations than typically investigated in clinical trials. The populations in the latter tend to be selected based on prior evidence of anti-proliferative or anti-tumour responses and favourable pharmacodynamics and pharmacokinetics in pre-clinical studies and early-stage trials (23). For example, trials investigating the efficacy of anti-PD-L1 immune checkpoint inhibitors for ovarian cancer treatment have largely restricted to treatment-naïve advanced stage (stage III-IV) epithelial ovarian cancer patients, but have not been successful (24-28) (Table 1, Supplementary Table 1).

Consequently, this analysis will not only enable evaluation of the efficacy of anti-PD-1 and anti-PD-L1 immune checkpoint inhibitors for the treatment of cancers where these medications are not currently approved, but also broader populations of patients with select cancers where these drugs have previously been approved for specific patient subgroups only (e.g., populations selected on the basis of treatment history, PD-L1 expression level, or tumour stage). This may provide evidence to prioritise further studies required for their approval and could identify repurposing opportunities even for cancers with existing immune checkpoint inhibitor indications.

For peer review only - http://bmjopen.bmj.com/site/about/guidelines.xhtml

Mendelian randomisation

Two-sample MR will be used to investigate the association between circulating PD-1 or PD-L1 protein expression levels and survival for each of the six cancer types separately. This will use measurements of genetic variant-exposure and genetic variant-outcome associations from non-overlapping samples representative of the same underlying population, permitting analyses to leverage large-scale genetic association data for protein measures and cancer survival (29, 30).

MR should be less vulnerable to conventional issues of confounding, as genetic germline variants are randomly assorted at meiosis (5, 29-35). As germline genetic variants are fixed and cannot be influenced by subsequent disease status, MR analyses are immune to reverse causation bias (5, 29-33). Since MR analyses often utilise existing genetic association data, causal relationships can be tested in a more cost-effective and time-efficient manner than in randomised controlled trials (1, 29-31, 33).

Objectives

The aim of this study will be to investigate the association between the on-target effects of anti-PD-1 and anti-PD-L1 immune checkpoint inhibitors and cancer survival using two-sample MR to identify potential drug repurposing opportunities.

METHODS AND ANALYSIS

Exposures

Single-nucleotide polymorphisms (SNPs) associated with circulating PD-1 or PD-L1 expression levels will be used to proxy expression of these proteins. These genetic instruments will be selected from a GWAS of circulating proteins in 54,306 participants of European ancestry in the UK Biobank cohort (36). Statistical analysis, imputation, quality control, and protein expression quantification in this study have been described previously (36).

Outcomes

Genetic association data will be obtained from GWAS of cancer survival in individuals of European ancestry with breast (37), colorectal (38), lung (unpublished), melanoma (39), ovarian (40) and prostate cancer (40). The outcome in each GWAS was defined as cancer-specific mortality, except for the lung cancer GWAS which defined the outcome as all-cause mortality and the ovarian cancer GWAS which examined both progression-free survival and overall survival (all-cause) as outcomes (37-41). Patients for each cancer type who participated in the Genomics England survival GWAS will be combined with the respective cancer survival GWAS to increase statistical power (Table 2).

For peer review only - http://bmjopen.bmj.com/site/about/guidelines.xhtml

Table 2: Number of patients and mortality events occurring in the site-specific genome-wide association study (GWAS) (37-41) and Genomics England GWAS for each cancer site. The lung and ovarian cancer mortality events are from all causes, whilst the mortality events for the other cancer sites are cancer site-specific.

Cancer site	Number of patients			Number of events		
	Site-specific GWAS	Genomics England	Total	Site-specific GWAS	Genomics England	Total
Breast	91,686 (37)	2,183	93,869	7,531 (37)	238	7,769
Colorectal	16,964 (38)	2,190	19,154	4,010 (38)	541	4,551
Lung	10,036 (unpublished)	1,318	11,354	6,088 (unpublished)	592	6,680
Melanoma	10,982 (39)	219	11,201	1,041 (39)	108	1,149
Ovarian	2,901 (40)	494	3,395	1,656*	217	1,873
Prostate	24,023 (41)	-**	24,023	3,513 (41)	-**	3,513

**The number of mortality events in the ovarian cancer GWAS was estimated based on the event rate of 11 OCAC studies (AUS, BAV, BEL, HAW, HSK, MAC, MAL, MAY, NCO, NEC, PVD) (0.571) (42) and the ovarian cancer GWAS sample size (2,901) (40).*
***There were fewer than 50 prostate cancer patients included in the Genomics England survival GWAS so these will not be combined with the site-specific prostate cancer GWAS.*

Data harmonisation
Harmonisation of genetic data is the process by which the exposure and outcome GWAS summary statistics are joined together and oriented to reflect the same effect alleles. Therefore, harmonised data will only include SNPs which were common to both the protein expression and respective cancer survival GWAS.

Harmonisation will be performed using the harmonise_data function from the TwoSampleMR R package (https://mrcieu.github.io/TwoSampleMR/) (43, 44). This will use the function's default option which infers the positive strand using allele frequencies for palindromic SNPs (https://mrcieu.github.io/TwoSampleMR/) (43, 44). The correlation between exposure and outcome GWAS SNP effect allele frequencies will be compared following data harmonisation. If data harmonisation has been successful, the correlation coefficient would be expected to be close to 1, as this would suggest that the same alleles have been chosen as the effect allele in both GWAS summary statistic datasets.

Mendelian randomisation
Assumptions
There are three key assumptions of MR: relevance, exchangeability, and exclusion restriction (32, 35). The relevance assumption states that the genetic instrument must be associated with the exposure of interest, for example in this study the expression level of the drugs' protein target (29, 35). The second MR assumption, exchangeability, requires there are no common causes of the instrument and outcome (29, 35). The final MR assumption, exclusion restriction, states that there must not be any horizontal pleiotropy (29, 31-34, 45). Horizontal

For peer review only - http://bmjopen.bmj.com/site/about/guidelines.xhtml

pleiotropy occurs when there are additional pathways between the instruments and outcome, independent of the exposure (29, 35, 45).

Genetic instrument selection

The UK Biobank protein expression GWAS summary statistics (36) will be used to select SNPs associated with circulating PD-1 or PD-L1 expression. The PD-1 and PD-L1 proteins are encoded by *PDCD1* (chr2:241849884 - 241858894 in human genome build 38 (hg38)) and *CD274* (chr9:5450503 – 5470566 in hg38), respectively.

To minimise vulnerability to horizontal pleiotropy, only SNPs within the coding region for the gene transcribing the target protein, known as *cis* SNPs, will be included in genetic instrument sets (45). *Cis* instruments to proxy both proteins will be constructed in PLINK version 1.9 (46, 47) using SNPs in or within 500 kilobases (kb) from *PDCD1* or *CD274* that are associated with expression of these proteins ($P<5x10^{-6}$) at linkage disequilibrium (LD) $r^2<0.30$ (based on clumping with a random sample of 10,000 European participants from the UK Biobank) (5, 45, 48).

Estimator

Where the genetic instrument consists of one SNP, the Wald ratio will be used to assess the association between a protein instrumented by this SNP and cancer survival (6, 33, 49). Where a genetic instrument consists of two or more SNPs, the inverse-variance weighted (IVW) method will instead be used to investigate the association between a protein instrumented by these SNPs and cancer survival (6, 33, 49). Any LD between SNPs included in an instrument will be accounted for in analysis using a SNP correlation matrix based on a random sample of 10,000 participants of European ancestry from the UK Biobank (35, 48, 50). Heterogeneity of MR results across independent SNPs included in the genetic instrument sets will be assessed using Cochran's Q tests and MR results will be compared across each SNP in the instrument by visual inspection (29, 33, 51).

The protein expression GWAS included age, sex, age-sex interaction terms, protein expression measurement batch, UK Biobank centre, genotyping array, the first twenty principal components (PCs) of genetic ancestry, and duration between blood sample collection and protein expression measurement as covariates (36). Aside from the breast cancer GWAS which did not adjust for any covariates, the cancer site-specific GWAS were all adjusted for genetic PCs (although for different numbers of PCs) (37-41). The colorectal cancer survival GWAS additionally adjusted for age at diagnosis, sex, genotyping platform and study where the data originated from (38). The lung cancer survival GWAS also adjusted for age and sex (unpublished). The melanoma survival GWAS included age and sex as covariates in addition to genotyping batch for one cohort (39). For the ovarian cancer survival GWAS, the primary study, residual disease, tumour stage, histology, tumour grade, and age were also adjusted for (40). For the ovarian cancer survival GWAS, the primary study, residual disease, tumour stage, histology, tumour grade, and age were also adjusted for (40). The prostate cancer survival GWAS additionally included age, diagnostic prostate specific antigen (PSA) level and Gleason score as covariates (41).

Power

Using the UK Biobank protein expression GWAS (36), the lead *cis* SNP for *PDCD1* (rs1011514130) explained approximately 2.97% of the variation in circulating PD-1 expression whilst the lead *cis* SNP for *CD274* (rs822340) explained approximately 4.83% of the variation in PD-L1 expression.

For peer review only - http://bmjopen.bmj.com/site/about/guidelines.xhtml

Across all six cancer types, there is an estimated power of 80% to detect hazard ratios of at least ≥ 1.61 or ≤ 0.62 per unit decrease in normalised protein expression levels (alpha set to 5%) (Table 3).

Table 3: Estimated number of participants (N), mortality event rate, median survival, and hazard ratio (HR) per standard deviation decrease detectable with 80% power for each cancer site.

Cancer	N	Event rate	Median survival (months)	HRs detectable at estimated 80% power	
				PD-1	PD-L1
Breast	93,869	0.083	64.8 (52)	HR ≥ 1.20<br>HR ≤ 0.83	HR ≥ 1.17<br>HR ≤ 0.86
Colorectal	19,154	0.238	38.4 (52)	HR ≥ 1.27<br>HR ≤ 0.78	HR ≥ 1.22<br>HR ≤ 0.82
Lung	11,354	0.588*	3.6 (52)	HR ≥ 1.22<br>HR ≤ 0.82	HR ≥ 1.18<br>HR ≤ 0.85
Melanoma	11,201	0.103	53.4 (53)	HR ≥ 1.61<br>HR ≤ 0.62	HR ≥ 1.50<br>HR ≤ 0.67
Ovarian	3,395	0.552*	30.1 (54)	HR ≥ 1.46<br>HR ≤ 0.68	HR ≥ 1.37<br>HR ≤ 0.73
Prostate	24,023	0.146	62.4 (52)	HR ≥ 1.32<br>HR ≤ 0.76	HR ≥ 1.26<br>HR ≤ 0.80

**All-cause mortality event rate.*
Hazard ratios (HR) per standard deviation decrease estimated to be detected at 80% power calculated with the survSNP R package (55) using the combined estimated sample size and event rate from each cancer survival GWAS and the respective Genomics England cancer survival GWAS, median survival, and assuming a false positive rate of 0.05.

Sensitivity analyses
The main analyses will be repeated using genetic instruments constructed with more stringent thresholds: significance P-value thresholds of 5 x 10^{-7} and 5 x 10^{-8}, window sizes of 250 kb and 100 kb on either side of the gene of interest, and LD r^2 thresholds of 0.2, 0.1, and 0.001. Although the primary analysis will only consider *cis* variants, *cis* and *trans* variants (> 500 kb from the gene of interest) will also be considered in secondary analyses. Instruments constructed from *cis* and t*rans* variants will be selected based on P-value and LD threshold ($P<5 \times 10^{-8}$, $r^2<0.001$) with reference to a random sample of 10,000 participants from the UK Biobank. Where instruments are constructed from two or more SNPs, the primary analysis will also be re-run iteratively excluding individual SNPs from instruments to investigate whether findings are driven by individual SNPs (35).

Colocalisation analysis will be performed using PWCoCo (https://github.com/jwr-git/pwcoco) to investigate whether any significant MR results are confounded due to LD between one variant causing a change in protein expression and another causing a change in cancer survival through an independent pathway (4, 49, 56, 57).

Pleiotropy will be investigated by conducting phenome-wide association studies (PheWAS) to investigate whether the genetic instruments proxying PD-1 or PD-L1 expression are also associated with other phenotypes. This will be achieved using MR Base (https://github.com/MRCIEU/TwoSampleMR) (43, 44). The significance thresholds will be Bonferroni-corrected for the number of traits looked up (30, 49). Although these methods will

For peer review only - http://bmjopen.bmj.com/site/about/guidelines.xhtml

not specifically investigate horizontal pleiotropy, they will assess the possible extent of either vertical pleiotropy or horizontal pleiotropy. Vertical pleiotropy occurs when there is a mediator in the pathway between the exposure and outcome and, in contrast to horizontal pleiotropy, does not violate MR assumptions (31, 35, 45, 56).

Index event bias (also known as collider bias) may occur in studies of cancer survival if the hypothesised causal factor being evaluated (in this case, PD-1 or PD-L1) is a risk factor for disease onset (4, 30, 58). If SNPs found to be significantly associated with cancer survival are also associated with risk of the same cancer type, methods including the SlopeHunter R package (https://github.com/Osmahmoud/SlopeHunter) will be used to evaluate and account for index event bias (59, 60).

Secondary analyses
Where feasible, sub-group analyses will be performed to explore the impact of treatment history and tumour stage on the findings. We expect hazard ratios to be larger in earlier tumour stages and in treatment-naive patients, compared to late-stage diagnoses and heavily treated patients, respectively.

Software
The TwoSampleMR R package (https://mrcieu.github.io/TwoSampleMR/) (43, 44) will be used to perform two-sample MR using summary-level data.

Patient and public involvement
Members of an existing group of cancer patients and caregivers volunteered to discuss this research project after a proposal had been drafted. The importance of potential side effects of medications and generalisability of findings to populations of non-European ancestry were highlighted. The concerns related to side effects will not be addressed here as they are outside of the scope of this analysis but will be considered as limitations and could be studied in the future using alternate data sources such as electronic medical record data. The analyses may be able to be performed for non-European populations if genetic survival data from cancer patients of non-European ancestry are available. Patients and the public were not involved in the design of this study.

ETHICS AND DISSEMINATION

The protein expression GWAS conducted by Sun et al. (2022) utilised UK Biobank data obtained under the approved application numbers 65851, 20361, 26041, 44257, 53639, 69804 (36).

The breast cancer survival GWAS conducted by Morra et al. (2021) followed the Declaration of Helsinki principles (37). The colorectal cancer survival GWAS conducted by Labadie et al. (2022) gained approval by the Fred Hutchinson Cancer Research Center Institutional Review Board (38). The lung cancer survival GWAS ethical approval information is unpublished. The melanoma survival GWAS conducted by Seviiri et al. (2022) gained approval by the Sydney Local Health District Ethics Review Committee (MIA cohort), the United Kingdom's National North West Multi-Centre Research Ethics Committee (UK Biobank cohort) and the Human Research Ethics Committee of QIMR Berghofer Medical Research Institute (protocol) (39). The ovarian cancer survival GWAS conducted by Johnatty et al. (2015) and the prostate cancer survival GWAS conducted by Szulkin et al. (2015) included primary

For peer review only - http://bmjopen.bmj.com/site/about/guidelines.xhtml

GWAS which individually gained ethical approval from human research ethics committees (40, 41).(37). The colorectal cancer survival GWAS conducted by Labadie et al. (2022) gained approval by the Fred Hutchinson Cancer Research Center Institutional Review Board (38). The lung cancer survival GWAS ethical approval information is unpublished. The melanoma survival GWAS conducted by Seviiri et al. (2022) gained approval by the Sydney Local Health District Ethics Review Committee (MIA cohort), the United Kingdom's National North West Multi-Centre Research Ethics Committee (UK Biobank cohort) and the Human Research Ethics Committee of QIMR Berghofer Medical Research Institute (protocol) (39). The ovarian cancer survival GWAS conducted by Johnatty et al. (2015) and the prostate cancer survival GWAS conducted by Szulkin et al. (2015) included primary GWAS which individually gained ethical approval from human research ethics committees (40, 41).

All participants involved in the protein expression GWAS and breast, colorectal, melanoma, ovarian (OCAC), and prostate cancer survival GWAS provided informed consent (36-41, 61).

The results of these analyses will be published and disseminated to members of the University of Bristol MRC Integrative Epidemiology Unit ICEP User Reference Group which is comprised of cancer patients and caregivers. Any statistical code will be made publicly available.

REFERENCES

1. Walker VM, Davey Smith G, Davies NM, Martin RM. Mendelian randomization: a novel approach for the prediction of adverse drug events and drug repurposing opportunities. Int J Epidemiol. 2017;46(6):2078-89.
2. Tran AA, Prasad V. Drug repurposing for cancer treatments: a well-intentioned, but misguided strategy. The Lancet Oncology. 2020;21(9):1134-6.
3. Zhang Z, Zhou L, Xie N, Nice EC, Zhang T, Cui Y, et al. Overcoming cancer therapeutic bottleneck by drug repurposing. Signal Transduct Target Ther. 2020;5(1):113.
4. Joharatnam-Hogan N, Alexandre L, Yarmolinsky J, Lake B, Capps N, Martin RM, et al. Statins as Potential Chemoprevention or Therapeutic Agents in Cancer: a Model for Evaluating Repurposed Drugs. Curr Oncol Rep. 2021;23(3):29.
5. Walker VM, Kehoe PG, Martin RM, Davies NM. Repurposing antihypertensive drugs for the prevention of Alzheimer's disease: a Mendelian randomization study. International Journal of Epidemiology. 2019;49(4):1132-40.
6. Liu J, Cheng Y, Li M, Zhang Z, Li T, Luo X-J. Genome-wide Mendelian randomization identifies actionable novel drug targets for psychiatric disorders. Neuropsychopharmacology. 2023;48(2):270-80.
7. Sung H, Ferlay J, Siegel RL, Laversanne M, Soerjomataram I, Jemal A, et al. Global Cancer Statistics 2020: GLOBOCAN Estimates of Incidence and Mortality Worldwide for 36 Cancers in 185 Countries. CA: A Cancer Journal for Clinicians. 2021;71(3):209-49.
8. Hanusova V, Skalova L, Kralova V, Matouskova P. Potential anti-cancer drugs commonly used for other indications. Curr Cancer Drug Targets. 2015;15(1):35-52.
9. Yang EJ, Wu C, Liu Y, Lv J, Sup Shim J. Revisiting Non-Cancer Drugs for Cancer Therapy. Curr Top Med Chem. 2016;16(19):2144-55.
10. Marin-Acevedo JA, Kimbrough EO, Lou Y. Next generation of immune checkpoint inhibitors and beyond. Journal of Hematology & Oncology. 2021;14(1):45.

For peer review only - http://bmjopen.bmj.com/site/about/guidelines.xhtml

11. Hanahan D. Hallmarks of Cancer: New Dimensions. Cancer Discovery. 2022;12(1):31-46.
12. Jeong T-J, Lee H-T, Gu N, Jang Y-J, Choi S-B, Park U-B, et al. The High-Resolution Structure Reveals Remarkable Similarity in PD-1 Binding of Cemiplimab and Dostarlimab, the FDA-Approved Antibodies for Cancer Immunotherapy. Biomedicines. 2022;10(12):3154.
13. Singh V, Sheikh A, Abourehab MAS, Kesharwani P. Dostarlimab as a Miracle Drug: Rising Hope against Cancer Treatment. Biosensors (Basel). 2022;12(8).
14. Wesolowski J, Tankiewicz-Kwedlo A, Pawlak D. Modern Immunotherapy in the Treatment of Triple-Negative Breast Cancer. Cancers (Basel). 2022;14(16).
15. Fessas P, Lee H, Ikemizu S, Janowitz T. A molecular and preclinical comparison of the PD-1-targeted T-cell checkpoint inhibitors nivolumab and pembrolizumab. Semin Oncol. 2017;44(2):136-40.
16. Medicines and Healthcare products Regulatory Agency. LIBTAYO: SUMMARY OF PRODUCT CHARACTERISTICS 2022 [Available from: https://mhraproducts4853.blob.core.windows.net/docs/1f0f40fa2f9461bd438b181736a620032651f70a.
17. Medicines and Healthcare products Regulatory Agency. JEMPERLI: SUMMARY OF PRODUCT CHARACTERISTICS 2022 [Available from: https://mhraproducts4853.blob.core.windows.net/docs/17d1049c69f319d0b5d3b392264f1473da412992.
18. Medicines and Healthcare products Regulatory Agency. OPDIVO: SUMMARY OF PRODUCT CHARACTERISTICS 2022 [Available from: https://mhraproducts4853.blob.core.windows.net/docs/8355d7f28eb4601b4f0cfd41f6b423cc0ecbb7ac.
19. Medicines and Healthcare products Regulatory Agency. KEYTRUDA: SUMMARY OF PRODUCT CHARACTERISTICS 2022 [Available from: https://mhraproducts4853.blob.core.windows.net/docs/9a01eb070d0f4b699fc009e2a0932ae62586477f.
20. Medicines and Healthcare products Regulatory Agency. Tecentriq: SUMMARY OF PRODUCT CHARACTERISTICS 2022 [Available from: https://mhraproducts4853.blob.core.windows.net/docs/9ee66a803927d06cf73407425f3c46aa9172e3d9.
21. Medicines and Healthcare products Regulatory Agency. Bavencio: SUMMARY OF PRODUCT CHARACTERISTICS 2023 [Available from: https://mhraproducts4853.blob.core.windows.net/docs/67a96f2b38c91da858ddea961907c4c8315b36ec.
22. Medicines and Healthcare products Regulatory Agency. IMFINZI: SUMMARY OF PRODUCT CHARACTERISTICS 2023 [Available from: https://mhraproducts4853.blob.core.windows.net/docs/c631685fa069c1e6b3c414043180b30082374d65.
23. Wong CC, Cheng KW, Rigas B. Preclinical predictors of anticancer drug efficacy: critical assessment with emphasis on whether nanomolar potency should be required of candidate agents. J Pharmacol Exp Ther. 2012;341(3):572-8.
24. Morand S, Devanaboyina M, Staats H, Stanbery L, Nemunaitis J. Ovarian Cancer Immunotherapy and Personalized Medicine. Int J Mol Sci. 2021;22(12).
25. Shoji T, Sato C, Tomabechi H, Takatori E, Kaido Y, Nagasawa T, et al. Expectations and Challenges of First-Line Maintenance Therapy for Advanced Ovarian Cancer. Medicina (Kaunas). 2021;57(5).
26. Ledermann JA, Colombo N, Oza AM, Fujiwara K, Birrer MJ, Randall LM, et al. Avelumab in combination with and/or following chemotherapy vs chemotherapy alone in

For peer review only

patients with previously untreated epithelial ovarian cancer: Results from the phase 3 javelin ovarian 100 trial. Gynecologic Oncology. 2020;159:13-4.
27. Moore KN, Bookman M, Sehouli J, Miller A, Anderson C, Scambia G, et al. Atezolizumab, Bevacizumab, and Chemotherapy for Newly Diagnosed Stage III or IV Ovarian Cancer: Placebo-Controlled Randomized Phase III Trial (IMagyn050/GOG 3015/ENGOT-OV39). Journal of Clinical Oncology. 2021;39(17):1842-55.
28. Harter P, Bidziński M, Colombo N, Floquet A, Pérez MJR, Kim J-W, et al. DUO-O: A randomized phase III trial of durvalumab (durva) in combination with chemotherapy and bevacizumab (bev), followed by maintenance durva, bev and olaparib (olap), in newly diagnosed advanced ovarian cancer patients. Journal of Clinical Oncology. 2019;37(15_suppl):TPS5598-TPS.
29. Zheng J, Baird D, Borges MC, Bowden J, Hemani G, Haycock P, et al. Recent Developments in Mendelian Randomization Studies. Curr Epidemiol Rep. 2017;4(4):330-45.
30. Mokry LE, Zhou S, Guo C, Scott RA, Devey L, Langenberg C, et al. Interleukin-18 as a drug repositioning opportunity for inflammatory bowel disease: A Mendelian randomization study. Scientific Reports. 2019;9(1):9386.
31. Gill D, Georgakis MK, Walker VM, Schmidt AF, Gkatzionis A, Freitag DF, et al. Mendelian randomization for studying the effects of perturbing drug targets. Wellcome Open Res. 2021;6:16.
32. Khasawneh LQ, Al-Mahayri ZN, Ali BR. Mendelian randomization in pharmacogenomics: The unforeseen potentials. Biomedicine & Pharmacotherapy. 2022;150:112952.
33. Tang B, Wang Y, Jiang X, Thambisetty M, Ferrucci L, Johnell K, et al. Genetic Variation in Targets of Antidiabetic Drugs and Alzheimer Disease Risk. A Mendelian Randomization Study. 2022;99(7):e650-e9.
34. Acosta JN, Szejko N, Falcone GJ. Mendelian Randomization in Stroke: A Powerful Approach to Causal Inference and Drug Target Validation. Frontiers in Genetics. 2021;12.
35. Yarmolinsky J, Díez-Obrero V, Richardson TG, Pigeyre M, Sjaarda J, Paré G, et al. Genetically proxied therapeutic inhibition of antihypertensive drug targets and risk of common cancers: A mendelian randomization analysis. PLOS Medicine. 2022;19(2):e1003897.
36. Sun BB, Chiou J, Traylor M, Benner C, Hsu Y-H, Richardson TG, et al. Genetic regulation of the human plasma proteome in 54,306 UK Biobank participants. bioRxiv. 2022:2022.06.17.496443.
37. Morra A, Escala-Garcia M, Beesley J, Keeman R, Canisius S, Ahearn TU, et al. Association of germline genetic variants with breast cancer-specific survival in patient subgroups defined by clinic-pathological variables related to tumor biology and type of systemic treatment. Breast Cancer Research. 2021;23(1):86.
38. Labadie JD, Savas S, Harrison TA, Banbury B, Huang Y, Buchanan DD, et al. Genome-wide association study identifies tumor anatomical site-specific risk variants for colorectal cancer survival. Scientific Reports. 2022;12(1):127.
39. Seviiri M, Scolyer RA, Bishop DT, Newton-Bishop JA, Iles MM, Lo SN, et al. Higher polygenic risk for melanoma is associated with improved survival in a high ultraviolet radiation setting. Journal of Translational Medicine. 2022;20(1):403.
40. Johnatty SE, Tyrer JP, Kar S, Beesley J, Lu Y, Gao B, et al. Genome-wide Analysis Identifies Novel Loci Associated with Ovarian Cancer Outcomes: Findings from the Ovarian Cancer Association Consortium. Clin Cancer Res. 2015;21(23):5264-76.
41. Szulkin R, Karlsson R, Whitington T, Aly M, Gronberg H, Eeles RA, et al. Genome-wide association study of prostate cancer-specific survival. Cancer Epidemiol Biomarkers Prev. 2015;24(11):1796-800.

For peer review only - http://bmjopen.bmj.com/site/about/guidelines.xhtml

42. Nagle CM, Dixon SC, Jensen A, Kjaer SK, Modugno F, deFazio A, et al. Obesity and survival among women with ovarian cancer: results from the Ovarian Cancer Association Consortium. Br J Cancer. 2015;113(5):817-26.
43. Hemani G, Zheng J, Elsworth B, Wade KH, Haberland V, Baird D, et al. The MR-Base platform supports systematic causal inference across the human phenome. Elife. 2018;7.
44. Hemani G, Tilling K, Davey Smith G. Orienting the causal relationship between imprecisely measured traits using GWAS summary data. PLOS Genetics. 2017;13(11):e1007081.
45. Schmidt AF, Finan C, Gordillo-Marañón M, Asselbergs FW, Freitag DF, Patel RS, et al. Genetic drug target validation using Mendelian randomisation. Nature Communications. 2020;11(1):3255.
46. Chang CC, Chow CC, Tellier LC, Vattikuti S, Purcell SM, Lee JJ. Second-generation PLINK: rising to the challenge of larger and richer datasets. GigaScience. 2015;4(1).
47. Purcell S, Neale B, Todd-Brown K, Thomas L, Ferreira MA, Bender D, et al. PLINK: a tool set for whole-genome association and population-based linkage analyses. Am J Hum Genet. 2007;81(3):559-75.
48. Auton A, Brooks LD, Durbin RM, Garrison EP, Kang HM, Korbel JO, et al. A global reference for human genetic variation. Nature. 2015;526(7571):68-74.
49. Gaziano L, Giambartolomei C, Pereira AC, Gaulton A, Posner DC, Swanson SA, et al. Actionable druggable genome-wide Mendelian randomization identifies repurposing opportunities for COVID-19. Nature Medicine. 2021;27(4):668-76.
50. Burgess S, Zuber V, Valdes-Marquez E, Sun BB, Hopewell JC. Mendelian randomization with fine-mapped genetic data: Choosing from large numbers of correlated instrumental variables. Genet Epidemiol. 2017;41(8):714-25.
51. Porcu E, Rüeger S, Lepik K, Agbessi M, Ahsan H, Alves I, et al. Mendelian randomization integrating GWAS and eQTL data reveals genetic determinants of complex and clinical traits. Nature Communications. 2019;10(1):3300.
52. Alessy SA, Davies EA, Rawlinson J, Baker M, Lüchtenborg M. How representative are colorectal, lung, breast and prostate cancer patients responding to the National Cancer Patient Experience Survey (CPES) of the cancer registry population in England? A population-based case control study. BMJ Open. 2019;9(12):e034344.
53. Sandru A, Voinea S, Panaitescu E, Blidaru A. Survival rates of patients with metastatic malignant melanoma. J Med Life. 2014;7(4):572-6.
54. Cheeseman S, Levick B, Sopwith W, Fenton H, Nam EJ, Kim D, et al. Ovarian Real-World International Consortium (ORWIC): A multicentre, real-world analysis of epithelial ovarian cancer treatment and outcomes. Frontiers in Oncology. 2023;13.
55. Owzar K, Li Z, Cox N, Jung SH. Power and sample size calculations for SNP association studies with censored time-to-event outcomes. Genet Epidemiol. 2012;36(6):538-48.
56. Zheng J, Haberland V, Baird D, Walker V, Haycock PC, Hurle MR, et al. Phenome-wide Mendelian randomization mapping the influence of the plasma proteome on complex diseases. Nature Genetics. 2020;52(10):1122-31.
57. Robinson JW, Hemani G, Babaei MS, Huang Y, Baird DA, Tsai EA, et al. An efficient and robust tool for colocalisation: Pair-wise Conditional and Colocalisation (PWCoCo). bioRxiv. 2022:2022.08.08.503158.
58. Mitchell RE, Hartley A, Walker VM, Gkatzionis A, Yarmolinsky J, Bell JA, et al. Strategies to investigate and mitigate collider bias in genetic and Mendelian randomization studies of disease progression. medRxiv. 2022:2022.04.22.22274166.

For peer review only

59. Mahmoud O, Dudbridge F, Davey Smith G, Munafo M, Tilling K. A robust method for collider bias correction in conditional genome-wide association studies. Nature Communications. 2022;13(1):619.
60. Cai S, Hartley A, Mahmoud O, Tilling K, Dudbridge F. Adjusting for collider bias in genetic association studies using instrumental variable methods. Genet Epidemiol. 2022;46(5-6):303-16.
61. Schumacher FR, Berndt SI, Siddiq A, Jacobs KB, Wang Z, Lindstrom S, et al. Genome-wide association study identifies new prostate cancer susceptibility loci. Hum Mol Genet. 2011;20(19):3867-75.

AUTHORS' CONTRIBUTIONS

JY and PCH: Conceptualization, Methodology, Writing - Review & Editing, Supervision; RMM: Writing - Review & Editing, Supervision; TB: Writing - Original Draft.

FUNDING STATEMENT

RMM is a National Institute for Health Research Senior Investigator (NIHR202411). RMM, JY, and PCH are supported by a Cancer Research UK 25 (C18281/A29019) programme grant (the Integrative Cancer Epidemiology Programme). RMM is also supported by the NIHR Bristol Biomedical Research Centre which is funded by the NIHR (BRC-1215-20011) and is a partnership between University Hospitals Bristol and Weston NHS Foundation Trust and the University of Bristol. RMM is affiliated with the Medical Research Council Integrative Epidemiology Unit at the University of Bristol which is supported by the Medical Research Council (MC_UU_00011/1, MC_UU_00011/3, MC_UU_00011/6, and MC_UU_00011/4) and the University of Bristol. Department of Health and Social Care disclaimer: The views expressed are those of the author(s) and not necessarily those of the NHS, the NIHR or the Department of Health and Social Care.

TB is supported by grant MR/W006308/1 for the GW4 BIOMED MRC DTP, awarded to the Universities of Bath, Bristol, Cardiff and Exeter from the Medical Research Council (MRC)/UKRI.

COMPETING INTERESTS

RM, JY and PCH have received funding from Cancer Research UK. TB receives funding from the GW4 BIOMED MRC DTP.

For peer review only

SUPPLEMENTARY MATERIAL

Supplementary table 1: Medicines and Healthcare products Regulatory Agency indications of anti-programmed cell death protein 1 (PD-1) and anti-programmed cell death ligand 1 (PD-L1) monoclonal antibodies obtained 25th March 2023 (represents monotherapy indications and ** represents indications as part of combination therapy).*
Molecular markers: epidermal growth factor receptor gene (EGFR), anaplastic lymphoma kinase gene (ALK), c-ros oncogene 1 gene (ROS1), deficient mismatch repair (dMMR), microsatellite instability high (MSI-H), combined positive score (CPS), tumour proportion score (TPS).
Cancers: non-small cell lung cancer (NSCLC), triple-negative breast cancer (TNBC), small cell lung cancer (SCLC).

Target	Drug	Cancer					
		Breast	Colorectal	Lung	Melanoma	Ovarian	Prostate
PD-1	Cemiplimab (16)	-	-	• Metastatic or locally advanced PD-L1 ≥ 50% TPS NSCLC (*EGFR, ALK, ROS1* wild-type)*	-	-	-
	Dostarlimab (17)	-	-	-	-	-	-
	Nivolumab (18)	-	• dMMR/MSI-H colorectal cancer following chemotherapy**	• Metastatic NSCLC (*EGFR*, *ALK* wild type)**<br>• Metastatic or locally advanced NSCLC following chemotherapy*<br>• Resectable NSCLC**	• Metastatic or unresectable melanoma*,**<br>• Metastatic or lymph node-involved melanoma patients following complete resection*	-	-
	Pembrolizumab (19)	• Locally advanced or early-stage TNBC with high risk of recurrence*,**<br>• Metastatic or locally recurrent unresectable PD-L1 CPS ≥ 10 TNBC**	• Treatment-naïve metastatic dMMR/MSI-H colorectal cancer*<br>• Metastatic or unresectable dMMR/MSI-H colorectal cancer following prior treatment*	• Metastatic NSCLC with PD-L1 ≥ 50% TPS (*EGFR, ALK* wild-type)*,**<br>• Metastatic or locally advanced PD-L1 ≥ 1% TPS NSCLC following chemotherapy and	• Metastatic or unresectable melanoma*<br>• Completely resected stage IIB, IIC, III melanoma*	-	-

				targeted therapy (if applicable)*			
PD-L1	Atezolizumab (20)	• Metastatic or locally advanced PD-L1 $\geq$ 1% TPS TNBC**	-	• Completely resected stage II-IIIA NSCLC with PD-L1 $\geq$ 50% and no progression on prior chemotherapy*<br>• Metastatic non-squamous NSCLC**<br>• Metastatic NSCLC PD-L1 $\geq$ 50% TPS (*EGFR, ALK* wild-type)*<br>• Metastatic or locally advanced NSCLC following chemotherapy and targeted therapy (if applicable)*<br>• Treatment-naïve extensive stage SCLC**	-	-	-
	Avelumab (21)	-	-	-	-	-	-
	Durvalumab (22)	-	-	• Locally advanced unresectable PD-L1 $\geq$ 1% TPS NSCLC without progression on chemoradiation*<br>• Treatment-naïve extensive-stage SCLC**	-	-	-

For peer review only

BMJ Open

Investigating the association between genetically proxied circulating levels of immune checkpoint proteins and cancer survival: protocol for a Mendelian randomisation analysis

Journal:	*BMJ Open*
Manuscript ID	bmjopen-2023-075981.R1
Article Type:	Protocol
Date Submitted by the Author:	10-Oct-2023
Complete List of Authors:	Bate, Tessa; University of Bristol<br>Martin, Richard; University of Bristol, ; NIHR Bristol Biomedical Research Centre, University Hospitals Bristol and Weston NHS Foundation Trust and the University of Bristol<br>Yarmolinsky, James; University of Bristol; Imperial College London, Department of Epidemiology and Biostatistics, School of Public Health<br>Haycock, Philip; University of Bristol
**Primary Subject Heading**:	Oncology
Secondary Subject Heading:	Genetics and genomics
Keywords:	ONCOLOGY, Cancer genetics < GENETICS, Prognosis, THERAPEUTICS

SCHOLARONE™
Manuscripts

Investigating the association between genetically proxied circulating levels of immune checkpoint proteins and cancer survival: protocol for a Mendelian randomisation analysis

Tessa Bate[1,2], Richard M Martin[1-3], James Yarmolinsky [1,2,4]*, Philip C Haycock [1,2]*

[1] Medical Research Council Integrative Epidemiology Unit, University of Bristol
[2] Population Health Sciences, Bristol Medical School, University of Bristol
[3] NIHR Bristol Biomedical Research Centre, University Hospitals Bristol and Weston NHS Foundation Trust and the University of Bristol
[4] Department of Epidemiology and Biostatistics, School of Public Health, Imperial College London

*Contributed equally

Correspondence to:
Philip C Haycock: philip.haycock@bristol.ac.uk
James Yarmolinsky: j.yarmolinsky@imperial.ac.uk

For peer review only

ABSTRACT

Introduction

Compared to the traditional drug development pathway, investigating alternative uses for existing drugs (i.e., drug repurposing) requires substantially less time, cost, and resources. Immune checkpoint inhibitors are licensed for the treatment of certain breast, colorectal, head and neck, lung and melanoma cancers. These drugs target immune checkpoint proteins to reduce the suppression of T cell activation by cancer cells. As T cell suppression is a hallmark of cancer common across anatomical sites, we hypothesise that immune checkpoint inhibitors could be repurposed for the treatment of additional cancers beyond the ones already indicated.

Methods and analysis

We will use two-sample Mendelian randomisation to investigate the effect of genetically proxied levels of protein targets of two immune checkpoint inhibitors - programmed cell death protein 1 (PD-1) and programmed death ligand 1 (PD-L1) - on survival of seven cancer types (breast, colorectal, head and neck, lung, melanoma, ovarian, and prostate). Summary genetic association data will be obtained from prior genome-wide association studies of circulating protein levels and cancer survival in populations of European ancestry. Various sensitivity analyses will be performed to examine the robustness of findings to potential violations of Mendelian randomisation assumptions, collider bias and the impact of alternative genetic instrument construction strategies. The impact of treatment history and tumour stage on the findings will also be investigated using summary-level and individual-level genetic data where available.

Ethics and dissemination

No separate ethics approval will be required for these analyses as we will be using data from previously published genome-wide association studies which individually gained ethical approval and participant consent. Results from analyses will be submitted for publication in

an open access peer-reviewed journal and statistical code will be made freely available upon the completion of the analysis.

STRENGTHS AND LIMITATIONS OF THIS STUDY

- As germline genetic variants proxying circulating protein levels are randomly assorted at meiosis and fixed at conception, Mendelian randomisation analyses examining the effect of these proteins on cancer survival should be less prone to conventional issues of confounding and cannot be influenced by reverse causation bias.
- The use of a two-sample Mendelian randomisation framework will permit us to leverage large-scale genetic association data from separate samples, thus enhancing statistical power and precision of estimates.
- The generalisability of our findings to populations of non-European ancestry may be unclear.
- Mendelian randomisation analysis can only evaluate the on-target effects of immune checkpoint inhibitors.
- Sample sizes of genome-wide association studies of cancer survival are low in comparison to cancer risk, which will limit the statistical power of our analyses.

INTRODUCTION

Drug repurposing is the use of approved drugs for another indication (1, 2). The traditional development and testing pathway of candidate drugs is expensive and time-consuming, with an estimated cost of $2-3 billion USD and 13 years of research on average required for a chemical compound to be approved for use in clinical practice (3). In contrast, drugs that are tested for a repurposed use should already have demonstrated success in phase I trials for their original indication and thus their safety profiles for human use are known (3-6). Consequently, clinical testing for a repurposed use of a drug can begin at phase II trials, reducing associated time and resource requirements (3-6).

Despite advances in screening and treatment strategies, the number of people diagnosed with, and dying from cancer, continues to increase. Globally, there were estimated to be 19.3 million new cancer diagnoses and 10.0 million cancer deaths in 2020 (7). Seven cancer sites (breast, colorectal, head and neck, lung, melanoma skin, ovarian, and prostate cancer) were estimated to contribute to 48% of the incidence and 45% of mortality from all cancer sites globally in 2020 (7). In addition to the high burden of cancer, there are issues associated with currently available treatments such as development of resistance, severity of side effects, and lack of efficacy in some individuals (8). Identifying new strategies for the treatment of these high-burden cancers using drug repurposing could minimise the cost and patient involvement required for the assessment of their efficacy. Several drugs have been successfully repurposed for cancer treatment, including non-cancer drugs such as thalidomide which was originally developed to treat morning sickness in pregnancy but is now approved to treat multiple myeloma (3, 8, 9).

Shared hallmarks of cancer common to different cancer sites represent an opportunity for drug repurposing using approved drugs which target these mechanisms across multiple sites

For peer review only - http://bmjopen.bmj.com/site/about/guidelines.xhtml

(3). One such hallmark is the avoidance of immune destruction, which can be suppressed using immune checkpoint inhibitors (3, 10, 11). The first immune checkpoint inhibitor approved by the US Food and Drug Administration (FDA) was the anti-cytotoxic T-lymphocyte associated protein 4 (CTLA-4) monoclonal antibody, ipilimumab, for the treatment of melanoma in 2011 (10). Following the approval of ipilimumab, several other immune checkpoint inhibitors have also been approved for a range of cancer indications.

Two examples of immune checkpoint proteins which have been successfully targeted in cancer treatment are programmed cell death protein 1 (PD-1) and programmed cell death ligand 1 (PD-L1) (10). The anti-PD-1 monoclonal antibodies include cemiplimab (Libtayo), dostarlimab (Jemperli), nivolumab (Opdivo) and pembrolizumab (Keytruda), and the anti-PD-L1 monoclonal antibodies include atezolizumab (Tecentriq), avelumab (Bavencio) and durvalumab (Imfinzi) (10, 12). These seven immune checkpoint inhibitors have been approved by the Medicines and Healthcare products Regulatory Agency (MHRA) for specific cancer indications, including some indications for the seven cancer types detailed above (Table 1, Supplementary Table 1). Across anti-PD-1 immune checkpoint inhibitors, there are approved indications for the treatment of breast, colorectal, head and neck, and lung cancers and melanoma, whilst anti-PD-L1 immune checkpoint inhibitors have been approved for breast and lung cancer treatment (13-19) (Table 1, Supplementary Table 1).

The approvals for anti-PD-1 and anti-PD-L1 immune checkpoint inhibitors are not uniform across cancer sites, even for the drugs targeting the same immune checkpoint protein (Table 1). However, this may be explained by an absence, rather than failure, of comparable clinical trials for certain drugs within these drug target categories. For example, pembrolizumab is the only anti-PD-1 immune checkpoint inhibitor approved for a breast cancer indication by the MHRA (16) (Table 1), likely due to lack of complete late-stage clinical trials with large sample sizes investigating the efficacy of the other three anti-PD-1 immune checkpoint inhibitors (20, 21). Similarly, although nivolumab and pembrolizumab both have colorectal cancer indications, there are differences in the characteristics of the patient populations that they are approved to treat (15, 16) (Table 1, Supplementary Table 1). Nivolumab is approved to treat mismatch repair deficient (dMMR) or microsatellite instability high (MSI-H) colorectal cancer patients after chemotherapy as part of a combination therapy with ipilimumab (15) (Supplementary Table 1). In contrast, pembrolizumab as monotherapy is approved to treat metastatic or unresectable dMMR/MSI-H colorectal cancer, the latter following previous treatment (16) (Supplementary Table 1).

The approved indications of these immune checkpoint inhibitors are highly specific in many cases, particularly with respect to molecular tumour markers and treatment history. For example, durvalumab has been approved by the MHRA for the treatment of adult patients with locally advanced, unresectable non-small cell lung cancer if at least 1% of their tumour cells express PD-L1 and their disease did not advance after previous platinum-based chemoradiotherapy (19) (Supplementary Table 1). The populations in clinical trials tend to be selected based on prior evidence of anti-proliferative or anti-tumour responses and favourable pharmacodynamics and pharmacokinetics in pre-clinical studies and early-stage trials (22). For example, trials investigating the efficacy of anti-PD-L1 immune checkpoint inhibitors for ovarian cancer treatment have largely been restricted to evaluating treatment-naïve advanced stage (stage III-IV) epithelial ovarian cancer patients, but have not been successful (23-27) (Table 1, Supplementary Table 1).

For peer review only

Table 1. Medicines and Healthcare products Regulatory Agency (MHRA) indications of anti-programmed cell death protein 1 (PD-1) and anti-programmed cell death ligand 1 (PD-L1) monoclonal antibodies, obtained 25th March 2023

Protein target	Immune checkpoint inhibitor	Cancer						
		Breast	Colorectal	Head and neck	Lung	Melanoma	Ovarian	Prostate
PD-1	Cemiplimab (13)	-	-	-	A	-	-	-
	Dostarlimab (14)	-	-	-	-	-	-	-
	Nivolumab (15)	-	A	A	A	A	-	-
	Pembrolizumab (16)	A	A	A	A	A	-	-
PD-L1	Atezolizumab (17)	A	-	-	A	-	-	-
	Avelumab (18)	-	-	-	-	-	-	-
	Durvalumab (19)	-	-	-	A	-	-	-

"A" represents immune checkpoint inhibitors with at least one approved indication for the cancer type either as monotherapy or as part of combination therapy).

The interaction between PD-L1 on the surface of cancer cells and PD-1 on the surface of activated T cells suppresses further T cell activation (10, 20, 21, 28). Therefore, PD-1/L1 inhibitors suppress this interaction and so support T cell activation during anti-cancer immune responses (10, 12). Whilst PD-1 is largely expressed on immune cells, PD-L1 is expressed on a wider variety of non-haematopoietic cells, including tumour cells (29-32). Although there is uncertainty over the prognostic value of blood-based measures, previous studies have found that higher circulating (i.e. blood-based) PD-1 and PD-L1 levels are associated with poorer prognosis for patients diagnosed with cancer at different anatomical sites. For example, higher plasma soluble PD-1 and PD-L1 expression levels were associated with decreased progression-free survival for patients with advanced-stage high-grade serous ovarian cancer compared to those with lower PD-1 and PD-L1 expression (33). However, when accounting for other clinical factors in multivariable analyses, only soluble PD-L1 expression levels remained associated with progression-free survival for these patients (33). Higher circulating soluble PD-L1 expression was also associated with decreased overall and progression-free survival in a meta-analysis of patients with cancer at different anatomical sites, including non-small cell lung cancer and melanoma patients who had been treated with immunotherapy (34). In contrast, although low serum exosomal PD-L1 expression was associated with increased median overall survival for pancreatic ductal adenocarcinoma patients compared to those with high exosomal PD-L1 expression, there was little statistical evidence to support this observed difference (35). Therefore, even though the prognostic roles of circulating PD-1 and PD-L1 expression levels have not been fully determined, there is some evidence supporting an association between blood-based measures of these immune checkpoint proteins and cancer survival, and the mechanism of action of these drugs is mediated by T cells (33-35).

For peer review only - http://bmjopen.bmj.com/site/about/guidelines.xhtml

Mendelian randomisation

Mendelian randomisation (MR) investigates the association between an exposure and outcome using genetic variants associated with the exposure as a proxy for the exposure of interest (1). Two-sample MR uses measurements of genetic variant-exposure and genetic variant-outcome associations from separate studies, permitting analyses to leverage large-scale genetic association data for protein measures and cancer survival (36, 37).

MR should be less vulnerable to conventional issues of confounding, as genetic germline variants are randomly assorted at meiosis (5, 36-42). As germline genetic variants are fixed and cannot be influenced by subsequent disease status, MR analyses are immune to reverse causation bias (5, 36-40). Since MR analyses often utilise existing genetic association data, causal relationships can be tested in a more cost-effective and time-efficient manner than in randomised controlled trials (1, 36-38, 40).

Aims

The aim of this study is to investigate the association between genetically proxied PD-1 and PD-L1 protein levels and survival of seven cancer types (breast, colorectal, head and neck, lung, melanoma, ovarian, and prostate). These seven cancer sites have been chosen for inclusion as they have the most well-powered and accessible genome-wide association study (GWAS) survival data and make an important contribution to the overall global cancer burden.

These analyses will enable us to evaluate the repurposing potential of PD-1 and PD-L1 to new cancer indications. This will include potential repurposing to cancers without any existing approvals, as well as repurposing to new patient populations for cancers with some existing approvals. Previous MR studies have focused almost exclusively on causes of cancer risk. By including cancers with approved indications for PD-1 and PD-L1 inhibitors, which serves as a positive control, our analyses will also provide insight into the applicability of MR to studies of cancer survival.

METHODS AND ANALYSIS

Exposures

Rather than investigating the efficacy of specific immune checkpoint inhibitor compounds, their on-target effects will be proxied using genetic instruments which represent decreased circulating levels of their protein targets, PD-1 and PD-L1. Since our primary instruments will be based on studies in blood, we anticipate that our analyses may not fully proxy the mechanism of PD-1 and PD-L1 inhibitors in all biologically relevant tissues, an issue we will address in instrument validation analyses (see below).

Single-nucleotide polymorphisms (SNPs) associated with circulating PD-1 or PD-L1 expression levels will be used to proxy expression of these proteins. These genetic instruments will be selected from a GWAS of circulating proteins in 54,306 participants of European ancestry in the UK Biobank cohort (43). Statistical analysis, imputation, quality control, and protein expression quantification in this study have been described previously (43).

For peer review only

Outcomes

Genetic association data will be obtained from GWAS of cancer survival in individuals of European ancestry with breast (44), colorectal (45), head and neck (unpublished), lung (unpublished), melanoma (46), ovarian (47) and prostate cancer (47). The outcome in each GWAS was defined as cancer-specific mortality, except for the lung and head and neck cancer GWAS which defined the outcomes as all-cause mortality, and the ovarian cancer GWAS which examined both progression-free survival and overall survival (all-cause) as outcomes (44-48). To increase statistical power, we will combine the consortium site-specific GWAS with additional studies of cancer survival in Genomics England (unpublished) (Table 2).

Table 2. Number of patients and mortality events occurring in the site-specific consortium genome-wide association study (GWAS) and Genomics England GWAS for each cancer site

Cancer site	Number of patients			Number of events		
	Consortium GWAS	Genomics England	Total	Consortium GWAS	Genomics England	Total
Breast	91,686 (44)	2,183	93,869	7,531 (44)	238	7,769
Colorectal	16,964 (45)	2,190	19,154	4,010 (45)	541	4,551
Head and neck	10,000 (unpublished)	196	10,196	3,300 (unpublished)	74	3,374
Lung	10,036 (unpublished)	1,318	11,354	6,088 (unpublished)	592	6,680
Melanoma	10,982 (46)	219	11,201	1,041 (46)	108	1,149
Ovarian	2,901 (47)	494	3,395	1,656*	217	1,873
Prostate	24,023 (48)	-**	24,023	3,513 (48)	-**	3,513

The head and neck, lung and ovarian cancer mortality events are from all causes, whilst the mortality events for the other cancer sites are cancer site-specific.
*The number of mortality events in the ovarian cancer GWAS was estimated based on the event rate of 11 OCAC studies (AUS, BAV, BEL, HAW, HSK, MAC, MAL, MAY, NCO, NEC, PVD) (0.571) (49) and the ovarian cancer GWAS sample size (2,901) (47).
**There were fewer than 50 prostate cancer patients included in the Genomics England survival GWAS so these will not be combined with the site-specific prostate cancer GWAS.

Data harmonisation

Harmonisation of genetic data is the process by which the exposure and outcome GWAS summary statistics are joined together and oriented to reflect the same effect alleles. Therefore, harmonised data will only include SNPs which were common to both the protein expression and respective cancer survival GWAS.

Harmonisation will be performed using the harmonise_data function from the TwoSampleMR R package (https://mrcieu.github.io/TwoSampleMR/) (50, 51). This will use the function's default option which infers the positive strand using allele frequencies for palindromic SNPs (https://mrcieu.github.io/TwoSampleMR/) (50, 51). The correlation between exposure and outcome GWAS SNP effect allele frequencies will be compared following data harmonisation. If data harmonisation has been successful, the correlation

For peer review only - http://bmjopen.bmj.com/site/about/guidelines.xhtml

coefficient would be expected to be close to 1, as this would suggest that the same alleles have been chosen as the effect allele in both GWAS summary statistic datasets.

Mendelian randomisation

Assumptions

There are three key assumptions of MR: relevance, exchangeability, and exclusion restriction (39, 42). The relevance assumption states that the genetic instrument must be associated with the exposure of interest, for example in this study the circulating levels of the drugs' protein target (36, 42). The second MR assumption, exchangeability, requires there are no common causes of the instrument and outcome (36, 42). The final MR assumption, exclusion restriction, states that there must be no horizontal pleiotropy (36, 38-41, 52). Horizontal pleiotropy occurs when there are additional pathways between the instruments and outcome, independent of the exposure (36, 42, 52).

Genetic instrument selection

The UK Biobank protein expression GWAS summary statistics (43) will be used to select SNPs associated with circulating PD-1 or PD-L1 concentration. The genomic regions of the genes encoding PD-1 (*PDCD1*, chr2:241849884 – 241858894 in human genome build 38 (hg38)) and PD-L1 (*CD274*, chr9:5450503 – 5470566 in hg38) will be used to define *cis* and *trans* genetic instruments based on different window sizes.

To minimise vulnerability to horizontal pleiotropy, only SNPs within and in proximity to the gene encoding the target protein, known as *cis* SNPs, will be included in genetic instrument sets for the main analyses (52). *Cis* instruments to proxy both proteins will be constructed in PLINK version 1.9 (53, 54) using SNPs in or within 500 kilobases (kb) from *PDCD1* or *CD274* that are associated with expression of these proteins ($P<5x10^{-6}$) at linkage disequilibrium (LD) $r^2<0.30$ (based on clumping with a random sample of 10,000 European participants from the UK Biobank) (5, 52, 55).

Estimator

Where the genetic instrument consists of one SNP, the Wald ratio will be used to assess the association between a protein instrumented by this SNP and cancer survival (6, 40, 56). Where a genetic instrument consists of two or more SNPs, the inverse-variance weighted (IVW) method will instead be used to investigate the association between a protein instrumented by these SNPs and cancer survival (6, 40, 56). Any LD between SNPs included in an instrument will be accounted for in analysis using a SNP correlation matrix based on a random sample of 10,000 participants of European ancestry from the UK Biobank (42, 55, 57). Heterogeneity of MR results across independent SNPs included in the genetic instrument sets will be assessed using Cochran's Q tests and MR results will be compared across each SNP in the instrument by visual inspection (36, 40, 58).

The protein expression GWAS included age, sex, age-sex interaction terms, protein expression level measurement batch, UK Biobank centre, genotyping array, the first twenty principal components (PCs) of genetic ancestry, and duration between blood sample collection and protein expression measurement as covariates (43).

Aside from the breast cancer GWAS which did not adjust for any covariates, the cancer site-specific GWAS were all adjusted for genetic PCs (although for different numbers of PCs) (44-48). The colorectal cancer survival GWAS additionally adjusted for age at diagnosis, sex,

For peer review only - http://bmjopen.bmj.com/site/about/guidelines.xhtml

genotyping platform and study where the data originated from (45).The head and neck cancer GWAS which will be additionally adjusted for age, sex, stage at diagnosis (stratified as early or late stage), and cancer sub-type, The lung cancer survival GWAS also adjusted for age and sex, and separated participants into early-stage (stage I-II), late-stage (stage III-IV) and all stage analyses (unpublished). The melanoma survival GWAS included age and sex as covariates in addition to genotyping batch for one cohort (46). For the ovarian cancer survival GWAS, the primary study, residual disease, tumour stage, histology, tumour grade, and age were also adjusted for (47). The prostate cancer survival GWAS additionally included age, diagnostic prostate specific antigen (PSA) level and Gleason score as covariates (48).

Power

Using the UK Biobank protein expression GWAS (43), the lead *cis* SNP for *PDCD1* (rs1011514130) explained approximately 2.97% of the variation in circulating PD-1 expression level whilst the lead *cis* SNP for *CD274* (rs822340) explained approximately 4.83% of the variation in PD-L1 expression level.

Across all seven cancer types, there is an estimated power of 80% to detect hazard ratios of at least ≥ 1.61 or ≤ 0.62 per unit decrease in normalised protein expression levels (alpha set to 5%) (Table 3).

Table 3. Estimated number of participants (N), mortality event rate, median survival, and hazard ratio (HR) per standard deviation decrease detectable with 80% power for each cancer site

Cancer	N	Event rate	Median survival (months)	HRs detectable at estimated 80% power	
				PD-1	PD-L1
Breast	93,869	0.083	64.8 (59)	HR ≥ 1.20<br>HR ≤ 0.83	HR ≥ 1.17<br>HR ≤ 0.86
Colorectal	19,154	0.238	38.4 (59)	HR ≥ 1.27<br>HR ≤ 0.78	HR ≥ 1.22<br>HR ≤ 0.82
Head and neck	10,196	0.331*	54.3 (60)	HR ≥ 1.33<br>HR ≤ 0.75	HR ≥ 1.26<br>HR ≤ 0.79
Lung	11,354	0.588*	3.6 (59)	HR ≥ 1.22<br>HR ≤ 0.82	HR ≥ 1.18<br>HR ≤ 0.85
Melanoma	11,201	0.103	53.4 (61)	HR ≥ 1.61<br>HR ≤ 0.62	HR ≥ 1.50<br>HR ≤ 0.67
Ovarian	3,395	0.552*	30.1 (62)	HR ≥ 1.46<br>HR ≤ 0.68	HR ≥ 1.37<br>HR ≤ 0.73
Prostate	24,023	0.146	62.4 (59)	HR ≥ 1.32<br>HR ≤ 0.76	HR ≥ 1.26<br>HR ≤ 0.80

*All-cause mortality event rate.

Hazard ratios (HR) per standard deviation decrease estimated to be detected at 80% power calculated with the survSNP R package (63) (https://cran.r-project.org/web/packages/survSNP/index.html) using the combined estimated sample size and event rate from each cancer survival GWAS and the respective Genomics England cancer survival GWAS, median survival, and assuming a false positive rate of 0.05.

Positive controls

Positive control analyses investigate the association between the exposure of interest and an outcome which has already been observed to have a causal association with this exposure (64). This enables the reliability of the genetic instruments for such exposures to be validated

For peer review only

(65). For these analyses, the positive control outcomes will be survival for cancers at sites which PD-1 or PD-L1 inhibitors have been approved for treatment by the MHRA (i.e., breast, head and neck, colorectal, lung, and melanoma cancer survival) (Table 1). However, these analyses will be crude positive controls as these drugs are approved to treat highly specific patient populations (Supplementary Table 1), whereas the cancer survival data have been generated from broader patient populations.

Instrument validation

Our main analyses assume that SNPs associated with circulating PD-1/L1 protein expression level in the general population will have similar effects on protein levels in cancer cases and biologically relevant tissues (defined as those tissues responsible for the therapeutic benefit of PD-1/L1 inhibition). However, as PD-1 expression is upregulated due to T cell activation and PD-L1 expression is induced by inflammation and carcinogenesis, SNP-protein effects may differ between the general population and cancer cases (66, 67). Thus, we will compare the strength and direction of SNP-protein associations amongst cancer cases, participants without a cancer diagnosis and in samples broadly representative of the general population in UK Biobank (68). We will perform these analyses for UK Biobank cancer cases pooled across the seven cancer sites which our analyses focus on (N = 3,375), each of the seven cancer sites individually (Table 4), and pooled across all cancer sites (68).

Table 4. Number of UK Biobank cancer cases and corresponding International Classification of Diseases tenth revision (ICD-10) code for each cancer site and with protein expression data available

Cancer site	ICD-10 code	N
Breast	C50	780
Colorectal	C18-20	545
Head and neck	C00-C14, C32	124
Lung	C34	391
Malignant melanoma	C43	292
Ovary	C56	89
Prostate	C61	1,154

Number of participants with each ICD-10 code obtained from Papier et al. (2023) (68).

The genetic instruments proxying circulating PD-1/L1 protein concentration will be further validated by investigating the strength and direction of the SNP-protein associations in UK Biobank cancer cases who were diagnosed with cancer prior to blood collection (prevalent cases) and those diagnosed with cancer following blood collection (incident cases). We will also assess whether the associations between these genetic instruments and circulating PD-1/L1 level differ by patient time-since-diagnosis. These sensitivity analyses will enable assessment as to whether the associations between these genetic instruments and circulating PD-1/L1 level (and so their strength as genetic instruments) differs for cancer patients over time.

Additionally, as PD-L1 is expressed by cells at a number of potentially biologically relevant tissues aside from blood, such as the tumour site, endothelial cells, and sites of metastases (66), we will also investigate the association between the constructed genetic instruments and expression of the gene encoding PD-L1, *CD274,* in these tissues.

For each cancer site of interest, we will explore the strength and direction of association between these genetic instruments and *CD274* expression in tumour samples obtained from

For peer review only - http://bmjopen.bmj.com/site/about/guidelines.xhtml

The Cancer Genome Atlas Program (TCGA) dataset (https://www.cancer.gov/tcga) (Table 5) to validate the instruments' strength in the biologically relevant target population and target tissue.

Table 5. Estimated number of tissue samples with germline genotype and gene expression data (N) available from The Cancer Genome Atlas Program (TCGA) dataset (https://www.cancer.gov/tcga) for each cancer site

Cancer site	Study name	Study abbreviation	N
Breast	Breast invasive carcinoma	BRCA	770
Colorectal	Colon adenocarcinoma	COAD	298
	Rectum adenocarcinoma	READ	109
	Total	-	407
Head and neck	Head and Neck squamous cell carcinoma	HNSC	366
Lung	Lung adenocarcinoma	LUAD	385
	Lung squamous cell carcinoma	LUSC	334
	Total	-	719
Melanoma	Skin Cutaneous Melanoma	SKCM	54
Ovarian	Ovarian serous cystadenocarcinoma	OV	211
Prostate	Prostate adenocarcinoma	PRAD	366

We will also investigate the strength and direction of these associations for each anatomical site using tissue sample data obtained from the Genotype-Tissue Expression (GTEx) database (https://www.gtexportal.org/home/) (Table 6) and for immune cell populations obtained from Database of Immune Cell Expression, Expression quantitative trait loci (eQTLs) and Epigenomics (DICE) (https://dice-database.org/). This will enable validation of the strength and direction of association of these instruments in the tissues of interest and further understanding of the background level of expression of this gene not specific to cancer biology.

Table 6. Estimated sample sizes (N) available for measurements of gene expression in tissues at each anatomical site of interest obtained from the Genotype-Tissue Expression (GTEx) version 8 dataset (https://www.gtexportal.org/home/tissue/)

Tissue site	GTEx tissue name	N
Breast	Breast – mammary tissue	396
Colorectal	Colon – sigmoid	318
	Colon – transverse	368
	Total	686
Head and neck	Minor salivary gland	144
Lung	Lung	515
Melanoma	Skin – not sun exposed (suprapubic)	517
	Sun – sun exposed (lower leg)	605
Ovarian	Ovary	167
Prostate	Prostate	221

For peer review only - http://bmjopen.bmj.com/site/about/guidelines.xhtml

Furthermore, as T cell level and function are affected by interactions between PD-1 and PD-L1, we will investigate the strength and direction of association of these genetic instruments with white blood cell count and function in general population samples as well as UK Biobank cancer cases and cancer-free participants (10, 12, 66, 69). This will serve as a positive control analysis, as we would expect that the genetic instruments proxying circulating PD-1/L1 levels will be associated with white blood cell metrics. Additionally, if the genetic instruments are associated with white blood cell metrics and cancer survival, this will provide evidence supporting a mechanism through which circulating PD-1/L1 level affects cancer survival mediated by white blood cells.

Overall, these validation analyses will enable assessment as to whether it is appropriate to assume that SNPs associated with circulating PD-1/L1 levels can also be used to proxy PD-L1 expression at multiple anatomical sites in cancer cases and in biologically relevant tissues.

Sensitivity analyses

The main analyses will be repeated using genetic instruments constructed with more stringent thresholds: significance P-value thresholds of $< 5 \times 10^{-7}$ and $< 5 \times 10^{-8}$, window sizes of 250 kb and 100 kb on either side of the gene of interest, and LD r^2 thresholds of 0.2, 0.1, and 0.001. Although the primary analysis will only consider *cis* variants, *cis* and *trans* variants (>500 kb from the gene of interest) will also be considered in secondary analyses. Instruments constructed from *cis* and *trans* variants will be selected based on P-value and LD threshold ($P<5 \times 10^{-8}$, $r^2<0.001$) with reference to a random sample of 10,000 participants from the UK Biobank. Where instruments are constructed from two or more SNPs, the primary analysis will also be re-run iteratively excluding individual SNPs from instruments to investigate whether findings are driven by individual SNPs (42).

Colocalisation analysis will be performed using Pair-Wise Conditional analysis and Colocalisation analysis (PWCoCo) (https://github.com/jwr-git/pwcoco) to investigate whether any significant MR results are biased due to LD between one variant causing a change in protein expression and another causing a change in cancer survival through an independent pathway (4, 56, 70, 71).

Pleiotropy will be investigated by conducting phenome-wide association studies (PheWAS) to investigate whether the genetic instruments proxying PD-1 or PD-L1 expression are also associated with other phenotypes. This will be achieved using the IEU Open GWAS project (https://gwas.mrcieu.ac.uk/) (50, 51). The significance thresholds will be Bonferroni-corrected for the number of traits looked up (37, 56). Although these methods will not specifically investigate horizontal pleiotropy, they will assess the possible extent of either vertical pleiotropy or horizontal pleiotropy. Vertical pleiotropy occurs when there is a mediator in the pathway between the exposure and outcome and, in contrast to horizontal pleiotropy, does not violate MR assumptions (38, 42, 52, 70).

Index event bias (also known as collider bias) may occur in studies of cancer survival if the hypothesised causal factor being evaluated for disease prognosis (in this case, PD-1 or PD-L1) is also a risk factor for disease onset (4, 37, 72). If SNPs found to be significantly associated with cancer survival are also associated with risk of the same cancer type, methods including the SlopeHunter R package (https://github.com/Osmahmoud/SlopeHunter) will be used to evaluate and account for index event bias (73, 74).

For peer review only - http://bmjopen.bmj.com/site/about/guidelines.xhtml

Secondary analyses

Where feasible, sub-group analyses will be performed to explore the impact of treatment history and tumour stage on the findings. We expect hazard ratios to be larger in earlier tumour stages and in treatment-naive patients, compared to late-stage diagnoses and heavily treated patients, respectively.

Software

The TwoSampleMR R package (https://mrcieu.github.io/TwoSampleMR/) (50, 51) will be used to perform two-sample MR using summary-level data.

Study status

Following submission of this protocol for publication, we have started to implement the analysis plan using breast, lung, melanoma and ovarian cancer survival as the outcomes of interest. We anticipate completing these analyses in 2024 and submitting a paper detailing the findings of these analyses for publication by the end of 2024.

Patient and public involvement

Members of an existing group of cancer patients and caregivers volunteered to discuss this research project after a proposal had been drafted. The importance of potential side effects of medications and generalisability of findings to populations of non-European ancestry were highlighted. The concerns related to side effects will not be addressed here as they are outside of the scope of this analysis but will be considered as limitations and could be studied in the future using alternate data sources such as electronic medical record data. The analyses may be able to be performed for non-European populations if genetic survival data from cancer patients of non-European ancestry are available. Patients and the public were not involved in the design of this study.

ETHICS AND DISSEMINATION

No separate ethics approval will be required for these analyses as we will be using data from previously published genome-wide association studies which individually gained ethical approval and participant consent.

The protein expression GWAS conducted by Sun et al. (2022) utilised UK Biobank data obtained under the approved application numbers 65851, 20361, 26041, 44257, 53639, 69804 (43).

The breast cancer survival GWAS conducted by Morra et al. (2021) followed the Declaration of Helsinki principles (44). The colorectal cancer survival GWAS conducted by Labadie et al. (2022) gained approval by the Fred Hutchinson Cancer Research Center Institutional Review Board (45). The head and neck cancer and lung cancer survival GWAS ethical approval information are unpublished. The melanoma survival GWAS conducted by Seviiri et al. (2022) gained approval by the Sydney Local Health District Ethics Review Committee (MIA cohort), the United Kingdom's National North West Multi-Centre Research Ethics Committee (UK Biobank cohort) and the Human Research Ethics Committee of QIMR Berghofer Medical Research Institute (protocol) (46). The ovarian cancer survival GWAS conducted by Johnatty et al. (2015) and the prostate cancer survival GWAS conducted by

For peer review only - http://bmjopen.bmj.com/site/about/guidelines.xhtml

Szulkin et al. (2015) included primary GWAS which individually gained ethical approval from human research ethics committees (47, 48).(44). The colorectal cancer survival GWAS conducted by Labadie et al. (2022) gained approval by the Fred Hutchinson Cancer Research Center Institutional Review Board (45). The melanoma survival GWAS conducted by Seviiri et al. (2022) gained approval by the Sydney Local Health District Ethics Review Committee (MIA cohort), the United Kingdom's National North West Multi-Centre Research Ethics Committee (UK Biobank cohort) and the Human Research Ethics Committee of QIMR Berghofer Medical Research Institute (protocol) (46). The ovarian cancer survival GWAS conducted by Johnatty et al. (2015) and the prostate cancer survival GWAS conducted by Szulkin et al. (2015) included primary GWAS which individually gained ethical approval from human research ethics committees (47, 48).

All participants involved in the protein expression GWAS and breast, colorectal, melanoma, ovarian (OCAC), and prostate cancer survival GWAS provided informed consent (43-48, 75).

The results of these analyses will be published and disseminated to members of the University of Bristol MRC Integrative Epidemiology Unit ICEP User Reference Group, which is comprised of cancer patients and caregivers. The results will also be submitted to an open access peer-reviewed journal for publication and any statistical code will be made publicly available.

CONTRIBUTORS

JY and PCH: Conceptualization, Methodology, Writing - Review & Editing, Supervision; RMM: Writing - Review & Editing, Supervision; TB: Writing - Original Draft.

FUNDING

TB is supported by grant MR/W006308/1 for the GW4 BIOMED MRC DTP, awarded to the Universities of Bath, Bristol, Cardiff and Exeter from the Medical Research Council (MRC)/UKRI. RMM is a National Institute for Health Research Senior Investigator (NIHR202411). RMM, JY, and PCH are supported by a Cancer Research UK 25 (C18281/A29019) programme grant (the Integrative Cancer Epidemiology Programme). RMM is also supported by the NIHR Bristol Biomedical Research Centre which is funded by the NIHR (BRC-1215-20011) and is a partnership between University Hospitals Bristol and Weston NHS Foundation Trust and the University of Bristol. RMM is affiliated with the Medical Research Council Integrative Epidemiology Unit at the University of Bristol which is supported by the Medical Research Council (MC_UU_00011/1, MC_UU_00011/3, MC_UU_00011/6, and MC_UU_00011/4) and the University of Bristol. Department of Health and Social Care disclaimer: The views expressed are those of the author(s) and not necessarily those of the NHS, the NIHR or the Department of Health and Social Care. JY is also supported by the NIHR Imperial Biomedical Research Centre (BRC).

COMPETING INTERESTS

RMM, JY and PCH have received funding from Cancer Research UK. TB receives funding from the GW4 BIOMED MRC DTP.

For peer review only - http://bmjopen.bmj.com/site/about/guidelines.xhtml

REFERENCES

1. Walker VM, Davey Smith G, Davies NM, Martin RM. Mendelian randomization: a novel approach for the prediction of adverse drug events and drug repurposing opportunities. Int J Epidemiol. 2017;46(6):2078-89.
2. Tran AA, Prasad V. Drug repurposing for cancer treatments: a well-intentioned, but misguided strategy. The Lancet Oncology. 2020;21(9):1134-6.
3. Zhang Z, Zhou L, Xie N, Nice EC, Zhang T, Cui Y, et al. Overcoming cancer therapeutic bottleneck by drug repurposing. Signal Transduct Target Ther. 2020;5(1):113.
4. Joharatnam-Hogan N, Alexandre L, Yarmolinsky J, Lake B, Capps N, Martin RM, et al. Statins as Potential Chemoprevention or Therapeutic Agents in Cancer: a Model for Evaluating Repurposed Drugs. Curr Oncol Rep. 2021;23(3):29.
5. Walker VM, Kehoe PG, Martin RM, Davies NM. Repurposing antihypertensive drugs for the prevention of Alzheimer’s disease: a Mendelian randomization study. International Journal of Epidemiology. 2019;49(4):1132-40.
6. Liu J, Cheng Y, Li M, Zhang Z, Li T, Luo X-J. Genome-wide Mendelian randomization identifies actionable novel drug targets for psychiatric disorders. Neuropsychopharmacology. 2023;48(2):270-80.
7. Sung H, Ferlay J, Siegel RL, Laversanne M, Soerjomataram I, Jemal A, et al. Global Cancer Statistics 2020: GLOBOCAN Estimates of Incidence and Mortality Worldwide for 36 Cancers in 185 Countries. CA: A Cancer Journal for Clinicians. 2021;71(3):209-49.
8. Hanusova V, Skalova L, Kralova V, Matouskova P. Potential anti-cancer drugs commonly used for other indications. Curr Cancer Drug Targets. 2015;15(1):35-52.
9. Yang EJ, Wu C, Liu Y, Lv J, Sup Shim J. Revisiting Non-Cancer Drugs for Cancer Therapy. Curr Top Med Chem. 2016;16(19):2144-55.
10. Marin-Acevedo JA, Kimbrough EO, Lou Y. Next generation of immune checkpoint inhibitors and beyond. Journal of Hematology & Oncology. 2021;14(1):45.
11. Hanahan D. Hallmarks of Cancer: New Dimensions. Cancer Discovery. 2022;12(1):31-46.
12. Fessas P, Lee H, Ikemizu S, Janowitz T. A molecular and preclinical comparison of the PD-1-targeted T-cell checkpoint inhibitors nivolumab and pembrolizumab. Semin Oncol. 2017;44(2):136-40.
13. Medicines and Healthcare products Regulatory Agency. LIBTAYO: SUMMARY OF PRODUCT CHARACTERISTICS 2022 [Available from: https://mhraproducts4853.blob.core.windows.net/docs/1f0f40fa2f9461bd438b181736a620032651f70a.
14. Medicines and Healthcare products Regulatory Agency. JEMPERLI: SUMMARY OF PRODUCT CHARACTERISTICS 2022 [Available from: https://mhraproducts4853.blob.core.windows.net/docs/17d1049c69f319d0b5d3b392264f1473da412992.
15. Medicines and Healthcare products Regulatory Agency. OPDIVO: SUMMARY OF PRODUCT CHARACTERISTICS 2022 [Available from: https://mhraproducts4853.blob.core.windows.net/docs/8355d7f28eb4601b4f0cfd41f6b423cc0ecbb7ac.
16. Medicines and Healthcare products Regulatory Agency. KEYTRUDA: SUMMARY OF PRODUCT CHARACTERISTICS 2022 [Available from: https://mhraproducts4853.blob.core.windows.net/docs/9a01eb070d0f4b699fc009e2a0932ae62586477f.
17. Medicines and Healthcare products Regulatory Agency. Tecentriq: SUMMARY OF PRODUCT CHARACTERISTICS 2022 [Available from:

For peer review only

https://mhraproducts4853.blob.core.windows.net/docs/9ee66a803927d06cf73407425f3c46aa9172e3d9.
18. Medicines and Healthcare products Regulatory Agency. Bavencio: SUMMARY OF PRODUCT CHARACTERISTICS 2023 [Available from: https://mhraproducts4853.blob.core.windows.net/docs/67a96f2b38c91da858ddea961907c4c8315b36ec.
19. Medicines and Healthcare products Regulatory Agency. IMFINZI: SUMMARY OF PRODUCT CHARACTERISTICS 2023 [Available from: https://mhraproducts4853.blob.core.windows.net/docs/c631685fa069c1e6b3c414043180b30082374d65.
20. Singh V, Sheikh A, Abourehab MAS, Kesharwani P. Dostarlimab as a Miracle Drug: Rising Hope against Cancer Treatment. Biosensors (Basel). 2022;12(8).
21. Wesolowski J, Tankiewicz-Kwedlo A, Pawlak D. Modern Immunotherapy in the Treatment of Triple-Negative Breast Cancer. Cancers (Basel). 2022;14(16).
22. Wong CC, Cheng KW, Rigas B. Preclinical predictors of anticancer drug efficacy: critical assessment with emphasis on whether nanomolar potency should be required of candidate agents. J Pharmacol Exp Ther. 2012;341(3):572-8.
23. Morand S, Devanaboyina M, Staats H, Stanbery L, Nemunaitis J. Ovarian Cancer Immunotherapy and Personalized Medicine. Int J Mol Sci. 2021;22(12).
24. Shoji T, Sato C, Tomabechi H, Takatori E, Kaido Y, Nagasawa T, et al. Expectations and Challenges of First-Line Maintenance Therapy for Advanced Ovarian Cancer. Medicina (Kaunas). 2021;57(5).
25. Ledermann JA, Colombo N, Oza AM, Fujiwara K, Birrer MJ, Randall LM, et al. Avelumab in combination with and/or following chemotherapy vs chemotherapy alone in patients with previously untreated epithelial ovarian cancer: Results from the phase 3 javelin ovarian 100 trial. Gynecologic Oncology. 2020;159:13-4.
26. Moore KN, Bookman M, Sehouli J, Miller A, Anderson C, Scambia G, et al. Atezolizumab, Bevacizumab, and Chemotherapy for Newly Diagnosed Stage III or IV Ovarian Cancer: Placebo-Controlled Randomized Phase III Trial (IMagyn050/GOG 3015/ENGOT-OV39). Journal of Clinical Oncology. 2021;39(17):1842-55.
27. Harter P, Bidziński M, Colombo N, Floquet A, Pérez MJR, Kim J-W, et al. DUO-O: A randomized phase III trial of durvalumab (durva) in combination with chemotherapy and bevacizumab (bev), followed by maintenance durva, bev and olaparib (olap), in newly diagnosed advanced ovarian cancer patients. Journal of Clinical Oncology. 2019;37(15_suppl):TPS5598-TPS.
28. Jeong T-J, Lee H-T, Gu N, Jang Y-J, Choi S-B, Park U-B, et al. The High-Resolution Structure Reveals Remarkable Similarity in PD-1 Binding of Cemiplimab and Dostarlimab, the FDA-Approved Antibodies for Cancer Immunotherapy. Biomedicines. 2022;10(12):3154.
29. Hudson K, Cross N, Jordan-Mahy N, Leyland R. The Extrinsic and Intrinsic Roles of PD-L1 and Its Receptor PD-1: Implications for Immunotherapy Treatment. Frontiers in Immunology. 2020;11.
30. Qin W, Hu L, Zhang X, Jiang S, Li J, Zhang Z, et al. The Diverse Function of PD-1/PD-L Pathway Beyond Cancer. Frontiers in Immunology. 2019;10.
31. Khan M, Zhao Z, Arooj S, Fu Y, Liao G. Soluble PD-1: Predictive, Prognostic, and Therapeutic Value for Cancer Immunotherapy. Frontiers in Immunology. 2020;11.
32. Wang X, Yang X, Zhang C, Wang Y, Cheng T, Duan L, et al. Tumor cell-intrinsic PD-1 receptor is a tumor suppressor and mediates resistance to PD-1 blockade therapy. Proceedings of the National Academy of Sciences. 2020;117(12):6640-50.
33. Fanale D, Brando C, Corsini LR, Cutaia S, Di Donna MC, Randazzo U, et al. Low plasma PD-L1 levels, early tumor onset and absence of peritoneal carcinomatosis improve

For peer review only - http://bmjopen.bmj.com/site/about/guidelines.xhtml

prognosis of women with advanced high-grade serous ovarian cancer. BMC Cancer. 2023;23(1):437.

34. Scirocchi F, Strigari L, Di Filippo A, Napoletano C, Pace A, Rahimi H, et al. Soluble PD-L1 as a Prognostic Factor for Immunotherapy Treatment in Solid Tumors: Systematic Review and Meta-Analysis. Int J Mol Sci. 2022;23(22).

35. Park SJ, Park JY, Shin K, Hong TH, Lee M, Kim Y, et al. Clinical significance of serum-derived exosomal PD-L1 expression in patients with advanced pancreatic cancer. BMC Cancer. 2023;23(1):389.

36. Zheng J, Baird D, Borges MC, Bowden J, Hemani G, Haycock P, et al. Recent Developments in Mendelian Randomization Studies. Curr Epidemiol Rep. 2017;4(4):330-45.

37. Mokry LE, Zhou S, Guo C, Scott RA, Devey L, Langenberg C, et al. Interleukin-18 as a drug repositioning opportunity for inflammatory bowel disease: A Mendelian randomization study. Scientific Reports. 2019;9(1):9386.

38. Gill D, Georgakis MK, Walker VM, Schmidt AF, Gkatzionis A, Freitag DF, et al. Mendelian randomization for studying the effects of perturbing drug targets. Wellcome Open Res. 2021;6:16.

39. Khasawneh LQ, Al-Mahayri ZN, Ali BR. Mendelian randomization in pharmacogenomics: The unforeseen potentials. Biomedicine & Pharmacotherapy. 2022;150:112952.

40. Tang B, Wang Y, Jiang X, Thambisetty M, Ferrucci L, Johnell K, et al. Genetic Variation in Targets of Antidiabetic Drugs and Alzheimer Disease Risk. A Mendelian Randomization Study. 2022;99(7):e650-e9.

41. Acosta JN, Szejko N, Falcone GJ. Mendelian Randomization in Stroke: A Powerful Approach to Causal Inference and Drug Target Validation. Frontiers in Genetics. 2021;12.

42. Yarmolinsky J, Díez-Obrero V, Richardson TG, Pigeyre M, Sjaarda J, Paré G, et al. Genetically proxied therapeutic inhibition of antihypertensive drug targets and risk of common cancers: A mendelian randomization analysis. PLOS Medicine. 2022;19(2):e1003897.

43. Sun BB, Chiou J, Traylor M, Benner C, Hsu Y-H, Richardson TG, et al. Genetic regulation of the human plasma proteome in 54,306 UK Biobank participants. bioRxiv. 2022:2022.06.17.496443.

44. Morra A, Escala-Garcia M, Beesley J, Keeman R, Canisius S, Ahearn TU, et al. Association of germline genetic variants with breast cancer-specific survival in patient subgroups defined by clinic-pathological variables related to tumor biology and type of systemic treatment. Breast Cancer Research. 2021;23(1):86.

45. Labadie JD, Savas S, Harrison TA, Banbury B, Huang Y, Buchanan DD, et al. Genome-wide association study identifies tumor anatomical site-specific risk variants for colorectal cancer survival. Scientific Reports. 2022;12(1):127.

46. Seviiri M, Scolyer RA, Bishop DT, Newton-Bishop JA, Iles MM, Lo SN, et al. Higher polygenic risk for melanoma is associated with improved survival in a high ultraviolet radiation setting. Journal of Translational Medicine. 2022;20(1):403.

47. Johnatty SE, Tyrer JP, Kar S, Beesley J, Lu Y, Gao B, et al. Genome-wide Analysis Identifies Novel Loci Associated with Ovarian Cancer Outcomes: Findings from the Ovarian Cancer Association Consortium. Clin Cancer Res. 2015;21(23):5264-76.

48. Szulkin R, Karlsson R, Whitington T, Aly M, Gronberg H, Eeles RA, et al. Genome-wide association study of prostate cancer-specific survival. Cancer Epidemiol Biomarkers Prev. 2015;24(11):1796-800.

49. Nagle CM, Dixon SC, Jensen A, Kjaer SK, Modugno F, deFazio A, et al. Obesity and survival among women with ovarian cancer: results from the Ovarian Cancer Association Consortium. Br J Cancer. 2015;113(5):817-26.

For peer review only - http://bmjopen.bmj.com/site/about/guidelines.xhtml

50. Hemani G, Zheng J, Elsworth B, Wade KH, Haberland V, Baird D, et al. The MR-Base platform supports systematic causal inference across the human phenome. Elife. 2018;7.
51. Hemani G, Tilling K, Davey Smith G. Orienting the causal relationship between imprecisely measured traits using GWAS summary data. PLOS Genetics. 2017;13(11):e1007081.
52. Schmidt AF, Finan C, Gordillo-Marañón M, Asselbergs FW, Freitag DF, Patel RS, et al. Genetic drug target validation using Mendelian randomisation. Nature Communications. 2020;11(1):3255.
53. Chang CC, Chow CC, Tellier LC, Vattikuti S, Purcell SM, Lee JJ. Second-generation PLINK: rising to the challenge of larger and richer datasets. GigaScience. 2015;4(1).
54. Purcell S, Neale B, Todd-Brown K, Thomas L, Ferreira MA, Bender D, et al. PLINK: a tool set for whole-genome association and population-based linkage analyses. Am J Hum Genet. 2007;81(3):559-75.
55. Auton A, Brooks LD, Durbin RM, Garrison EP, Kang HM, Korbel JO, et al. A global reference for human genetic variation. Nature. 2015;526(7571):68-74.
56. Gaziano L, Giambartolomei C, Pereira AC, Gaulton A, Posner DC, Swanson SA, et al. Actionable druggable genome-wide Mendelian randomization identifies repurposing opportunities for COVID-19. Nature Medicine. 2021;27(4):668-76.
57. Burgess S, Zuber V, Valdes-Marquez E, Sun BB, Hopewell JC. Mendelian randomization with fine-mapped genetic data: Choosing from large numbers of correlated instrumental variables. Genet Epidemiol. 2017;41(8):714-25.
58. Porcu E, Rüeger S, Lepik K, Agbessi M, Ahsan H, Alves I, et al. Mendelian randomization integrating GWAS and eQTL data reveals genetic determinants of complex and clinical traits. Nature Communications. 2019;10(1):3300.
59. Alessy SA, Davies EA, Rawlinson J, Baker M, Lüchtenborg M. How representative are colorectal, lung, breast and prostate cancer patients responding to the National Cancer Patient Experience Survey (CPES) of the cancer registry population in England? A population-based case control study. BMJ Open. 2019;9(12):e034344.
60. Dittberner A, Friedl B, Wittig A, Buentzel J, Kaftan H, Boeger D, et al. Gender Disparities in Epidemiology, Treatment, and Outcome for Head and Neck Cancer in Germany: A Population-Based Long-Term Analysis from 1996 to 2016 of the Thuringian Cancer Registry. Cancers. 2020;12(11):3418.
61. Sandru A, Voinea S, Panaitescu E, Blidaru A. Survival rates of patients with metastatic malignant melanoma. J Med Life. 2014;7(4):572-6.
62. Cheeseman S, Levick B, Sopwith W, Fenton H, Nam EJ, Kim D, et al. Ovarian Real-World International Consortium (ORWIC): A multicentre, real-world analysis of epithelial ovarian cancer treatment and outcomes. Frontiers in Oncology. 2023;13.
63. Owzar K, Li Z, Cox N, Jung SH. Power and sample size calculations for SNP association studies with censored time-to-event outcomes. Genet Epidemiol. 2012;36(6):538-48.
64. Burgess S, Davey Smith G, Davies NM, Dudbridge F, Gill D, Glymour MM, et al. Guidelines for performing Mendelian randomization investigations. Wellcome Open Res. 2019;4:186.
65. Taschler B, Smith SM, Nichols TE. Causal inference on neuroimaging data with Mendelian randomisation. NeuroImage. 2022;258:119385.
66. Sharpe AH, Pauken KE. The diverse functions of the PD1 inhibitory pathway. Nature Reviews Immunology. 2018;18(3):153-67.
67. Kula A, Dawidowicz M, Kiczmer P, Prawdzic Seńkowska A, Świętochowska E. The role of genetic polymorphism within PD-L1 gene in cancer. Review. Experimental and Molecular Pathology. 2020;116:104494.

For peer review only

68. Papier K, Atkins JR, Tong TY, Gaitskell K, Desai T, Ogamba CF, et al. Identifying proteomic risk factors for cancer using prospective and exome analyses: 1,463 circulating proteins and risk of 19 cancers in the UK Biobank. medRxiv. 2023:2023.07.28.23293330.
69. Akbari P, Vuckovic D, Stefanucci L, Jiang T, Kundu K, Kreuzhuber R, et al. A genome-wide association study of blood cell morphology identifies cellular proteins implicated in disease aetiology. Nature Communications. 2023;14(1):5023.
70. Zheng J, Haberland V, Baird D, Walker V, Haycock PC, Hurle MR, et al. Phenome-wide Mendelian randomization mapping the influence of the plasma proteome on complex diseases. Nature Genetics. 2020;52(10):1122-31.
71. Robinson JW, Hemani G, Babaei MS, Huang Y, Baird DA, Tsai EA, et al. An efficient and robust tool for colocalisation: Pair-wise Conditional and Colocalisation (PWCoCo). bioRxiv. 2022:2022.08.08.503158.
72. Mitchell RE, Hartley A, Walker VM, Gkatzionis A, Yarmolinsky J, Bell JA, et al. Strategies to investigate and mitigate collider bias in genetic and Mendelian randomization studies of disease progression. medRxiv. 2022:2022.04.22.22274166.
73. Mahmoud O, Dudbridge F, Davey Smith G, Munafo M, Tilling K. A robust method for collider bias correction in conditional genome-wide association studies. Nature Communications. 2022;13(1):619.
74. Cai S, Hartley A, Mahmoud O, Tilling K, Dudbridge F. Adjusting for collider bias in genetic association studies using instrumental variable methods. Genet Epidemiol. 2022;46(5-6):303-16.
75. Schumacher FR, Berndt SI, Siddiq A, Jacobs KB, Wang Z, Lindstrom S, et al. Genome-wide association study identifies new prostate cancer susceptibility loci. Hum Mol Genet. 2011;20(19):3867-75.

For peer review only

For peer review only

For peer review only - http://bmjopen.bmj.com/site/about/guidelines.xhtml

SUPPLEMENTARY MATERIAL

Supplementary table 1: Medicines and Healthcare products Regulatory Agency indications of anti-programmed cell death protein 1 (PD-1) and anti-programmed cell death ligand 1 (PD-L1) monoclonal antibodies obtained 25th March 2023 (* represents monotherapy indications and ** represents indications as part of combination therapy).
Molecular markers: epidermal growth factor receptor gene (*EGFR*), anaplastic lymphoma kinase gene (*ALK*), c-ros oncogene 1 gene (*ROS1*), deficient mismatch repair (dMMR), microsatellite instability high (MSI-H), combined positive score (CPS), tumour proportion score (TPS).
Cancers: non-small cell lung cancer (NSCLC), head and neck squamous cell carcinoma (HNSCC), triple-negative breast cancer (TNBC), small cell lung cancer (SCLC).

Target	Drug	Cancer						
		Breast	Colorectal	Head and neck	Lung	Melanoma	Ovarian	Prostate
PD-1	Cemiplimab (13)	-	-	-	• Metastatic or locally advanced PD-L1 ≥ 50% TPS NSCLC (*EGFR*, *ALK*, *ROS1* wildtype)*	-	-	-
	Dostarlimab (14)	-	-	-	-	-	-	-
	Nivolumab (15)	-	• dMMR/MSI-H colorectal cancer following chemotherapy**	• Metastatic or recurrent HNSCC following prior treatment*	• Metastatic NSCLC (*EGFR*, *ALK* wild type)**<br>• Metastatic or locally advanced NSCLC following chemotherapy*<br>• Resectable NSCLC**	• Metastatic or unresectable melanoma*,**<br>• Metastatic or lymph node involved melanoma patients following complete resection*	-	-

For peer review only - http://bmjopen.bmj.com/site/about/guidelines.xhtml

	Pembrolizumab (16)	• Locally advanced or early-stage TNBC with high risk of recurrence*,**<br>• Metastatic or locally recurrent unresectable PD-L1 CPS ≥ 10 TNBC**	• Treatment-naïve metastatic dMMR/MSI-H colorectal cancer*<br>• Metastatic or unresectable dMMR/MSI-H colorectal cancer following prior treatment*	• Treatment-naïve metastatic or unresectable recurrent PD-L1 CPS ≥ 1 HNSCC*,**<br>• Metastatic or recurrent PD-L1 TPS ≥ 50% HNSCC following prior chemotherapy*	• Metastatic NSCLC with PD-L1 ≥ 50% TPS (*EGFR*, *ALK* wild-type)*,**<br>• Metastatic or locally advanced PD-L1 ≥ 1% TPS NSCLC following chemotherapy and targeted therapy (if applicable)*	•Metastatic or unresectable melanoma*<br>•Completely resected stage IIB, IIC, III melanoma*	-	-
PD-L1	Atezolizumab (17)	• Metastatic or locally advanced PD-L1 ≥ 1% TPS TNBC**	-	-	• Completely resected stage II-IIIA NSCLC with PD-L1 ≥ 50% and no progression on prior chemotherapy*<br>• Metastatic nonsquamous NSCLC**<br>• Metastatic NSCLC PD-L1 ≥ 50% TPS (*EGFR*, *ALK* wildtype)*<br>• Metastatic or locally advanced NSCLC following chemotherapy and targeted therapy (if applicable)*<br>• Treatment-naïve extensive stage SCLC**	-	-	-
	Avelumab (18)	-	-	-	-	-	-	-

For peer review only - http://bmjopen.bmj.com/site/about/guidelines.xhtml

	Durvalumab (19)	-	-	-	• Locally advanced unresectable PD-L1 ≥ 1% TPS NSCLC without progression on chemoradiation*<br>• Treatment-naïve extensive-stage SCLC**	-	-	-

For peer review only
